# Human Induced Pluripotent Stem Cell-Derived TDP-43 Mutant Neurons Exhibit Consistent Functional Phenotypes Across Multiple Gene Edited Lines Despite Transcriptomic and Splicing Discrepancies

**DOI:** 10.3389/fcell.2021.728707

**Published:** 2021-09-29

**Authors:** Alec S. T. Smith, Changho Chun, Jennifer Hesson, Julie Mathieu, Paul N. Valdmanis, David L. Mack, Byung-Ok Choi, Deok-Ho Kim, Mark Bothwell

**Affiliations:** ^1^Department of Physiology and Biophysics, University of Washington, Seattle, WA, United States; ^2^Institute for Stem Cell and Regenerative Medicine, University of Washington, Seattle, WA, United States; ^3^Department of Bioengineering, University of Washington, Seattle, WA, United States; ^4^Department of Comparative Medicine, University of Washington, Seattle, WA, United States; ^5^Division of Medical Genetics, University of Washington, Seattle, WA, United States; ^6^Department of Rehabilitation Medicine, University of Washington, Seattle, WA, United States; ^7^Department of Neurology, Samsung Medical Center, Sungkyunkwan University School of Medicine, Seoul, South Korea; ^8^Stem Cell and Regenerative Medicine Institute, Samsung Medical Center, Seoul, South Korea; ^9^Department of Health Sciences and Technology, The Samsung Advanced Institute for Health Sciences & Technology (SAIHST), Sungkyunkwan University, Seoul, South Korea; ^10^Department of Biomedical Engineering, Johns Hopkins University, Baltimore, MD, United States; ^11^Department of Medicine, Johns Hopkins University School of Medicine, Baltimore, MD, United States; ^12^Department of Neurology, Johns Hopkins University School of Medicine, Baltimore, MD, United States

**Keywords:** ALS (amyotrophic lateral sclerosis), iPSC (induced pluripotent stem cell), transcriptomics, electrophysiologic analysis, disease model

## Abstract

Gene editing technologies hold great potential to enhance our ability to model inheritable neurodegenerative diseases. Specifically, engineering multiple amyotrophic lateral sclerosis (ALS) mutations into isogenic cell populations facilitates determination of whether different causal mutations cause pathology *via* shared mechanisms, and provides the capacity to separate these mechanisms from genotype-specific effects. As gene-edited, cell-based models of human disease become more commonplace, there is an urgent need to verify that these models constitute consistent and accurate representations of native biology. Here, commercially sourced, induced pluripotent stem cell-derived motor neurons from Cellular Dynamics International, edited to express the ALS-relevant mutations TDP-43^M337V^ and TDP-43^Q331K^ were compared with in-house derived lines engineered to express the TDP-43^Q331K^ mutation within the WTC11 background. Our results highlight electrophysiological and mitochondrial deficits in these edited cells that correlate with patient-derived cells, suggesting a consistent cellular phenotype arising from TDP-43 mutation. However, significant differences in the transcriptomic profiles and splicing behavior of the edited cells underscores the need for careful comparison of multiple lines when attempting to use these cells as a means to better understand the onset and progression of ALS in humans.

## Introduction

Amyotrophic lateral sclerosis (ALS) is a clinically and genetically heterogeneous neurodegenerative disease with an incidence rate of roughly 1 in 50,000 people (Chiò et al., 2013). It manifests as degenerative changes (and eventual loss) of upper and lower motor neurons, leading to progressive muscular atrophy and weakness, increased fatigue, and problems with swallowing that typically result in respiratory failure and death. The condition is currently incurable and carries significant personal, societal, and economic burden (Lopez-Bastida et al., 2009; Schepelmann et al., 2010). Riluzole and Edaravone are the only drugs in the United States or Europe currently licensed for use in ALS patients but they are typically only capable of prolonging life by a few months (Bensimon et al., 1994; Petrov et al., 2017; Jackson et al., 2019; Shefner et al., 2019).

There is an urgent need to develop novel therapeutic options capable of ameliorating ALS symptoms or slowing disease progression to improve patient care and quality of life. It is envisioned that greater mechanistic understanding of ALS etiology will enable the identification of suitable biomarkers and therapeutic targets, which will in turn lead to the development of novel treatments. However, the clinical and genetic heterogeneity inherent to ALS pathophysiology makes accurate modeling of the disease difficult and reduces the predictive power of current preclinical animal and cell-based models (Benatar, 2007; Scott et al., 2008; Marangi and Traynor, 2015; Grad et al., 2017).

Induced pluripotent stem cell (iPSC)-based motor neuron models have significantly increased our capacity to model ALS, as well as other neurodegenerative diseases, in human cells (Liu and Deng, 2016; Pasteuning-Vuhman et al., 2020). Recent advances in gene editing techniques hold the potential to further expand these capabilities by enabling the study of phenotypic changes caused by multiple ALS-relevant mutations expressed against consistent background genotypes and in reference to isogenic controls (Heman-Ackah et al., 2016). Such models are now enabling detailed analysis of disease progression *in vitro*, as well as comparison of phenotypic differences across multiple causal mutations to isolate common ALS etiological features from mutation specific effects.

As promising as these technologies are, there remains a need to ensure that the gene editing strategies utilized to create ALS-relevant lines produce cells that are not only accurate representations of the human disease but also exhibit consistent phenotypes regardless of the editing strategy employed (Berry et al., 2018). This is particularly important for commercially sourced cells, that are likely to be more widely adopted than those created in academic labs, and certainly a favored option for drug companies due to ease of scaling and consistency considerations. To address these concerns, this manuscript aims to characterize the phenotype of commercially sourced motor neurons bearing two ALS-relevant mutations in the TDP-43 gene, *TARDBP*. Both the Q331K and the M337V variants were provided by Cellular Dynamics International (CDI) and were created using nuclease-mediated engineering that employed the same nuclease but was based on separate donor templates. Each mutant line was engineered from the same iPSC source used to produce their wild type iCell motor neuron line. The phenotypes of these cells were directly compared to that of an in house engineered line expressing the TDP-43^Q331K^ mutation in the WTC11 genotype. Analysis revealed consistent deficits in electrophysiological metrics, mitochondrial structure, and metabolic function in these engineered lines that correlated with results from patient-derived sources and previous studies. However, RNA-seq analysis revealed significant differences in gene expression profiles and splicing behavior between the commercial and in house developed mutant lines. Most notably, differences in stathmin-2 splice activity and *CHCHD2* expression patterns highlight the phenotypic inconsistency exhibited by these gene edited iPSC-derived motor neurons. The presence of significant transcriptomic differences between iPSC-derived motor neurons edited to express the same mutation in TDP-43 highlights the need for caution when attempting to define any observed phenotypes as representative of ALS cellular pathology.

## Results

### Establishment of TDP-43 Mutant Induced Pluripotent Stem Cell Lines and Confirmation of a Motor Neuron Phenotype in Differentiated Cells

**FIGURE 1:**
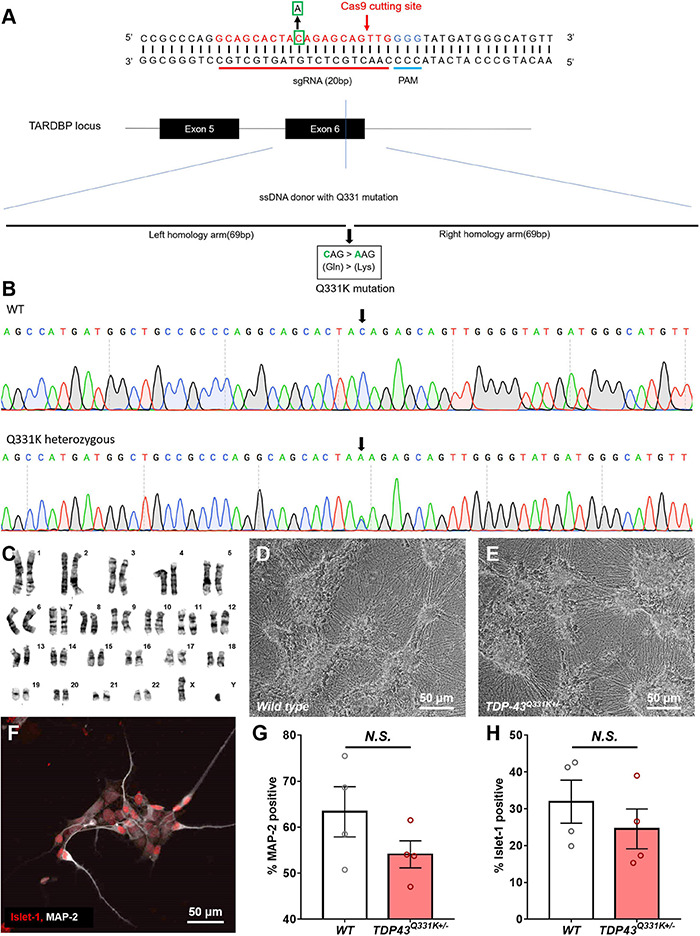
Production of a CRISPR-edited TDP-43^Q331K+/–^ mutant WTC11 iPSC line and its subsequent differentiation into motor neurons. **(A)** Schematic diagram of the genome editing strategy used to introduce a Q331K point mutation into the *TARDBP* locus of human WTC11 iPSCs. The sgRNA is marked in red, and the PAM sequence is presented in blue. The Cas9 cutting site in exon 6 of human *TARDBP* is indicated by a red arrow. The base C in the green square is replaced with an A, thus resulting in Gln331Lyss (CAG- > AAG), producing a single precision point mutation. The long sequence below is a single stranded DNA (ssDNA) donor with the desired mutation and homologous arms (left 69 bp and right 69 bp). **(B)** Sequencing data confirming the presence of a heterozygous point mutation (C- > A) in the examined WTC11 line, facilitating the Gln331Lys mutation in the translated amino acid sequence. **(C)** Karyotype analysis of our CRISPR-edited *TDP-43*^Q331K+/–^ WTC11 line, illustrating a normal male karyotype following gene editing. **(D,E)** Bright field images of wild type and *TDP-43*^Q331K+/–^ mutant motor neurons differentiated from WTC11 iPSCs. Images were collected at day 45 post-induction. **(F)** Immunocytochemical stain, illustrating positive staining for the motor neuron-specific marker Islet-1 in our human motor neurons differentiated in house from WTC11 iPSCs. **(G,H)** Percentage of MAP-2 and Islet-1 positive cells present in wild type and *TDP-43*^Q331K+/–^ mutant WTC11 populations subjected to our motor neuron differentiation protocol. Observed differences were not statistically significant between groups (N.S.). Analysis was performed at 26 days post-induction.

Cellular Dynamics International markets human iPSC-derived motor neurons bearing two distinct gene edited point mutations in the *TARDBP* locus, producing heterozygous TDP-43^M337V^ and TDP^Q331K^ genotypes (Supplementary Figure 1). In order to compare the phenotype of these cells to those subjected to a different CRISPR-based engineering strategy, a TDP-43^Q331K+/–^ mutation was engineered into the widely used WTC11 iPSC line (Kreitzer et al., 2013; Miyaoka et al., 2014). The Q331K variant was chosen in this instance as it generated a stronger phenotype in a transgenic mouse line than a similar transgenic line expressing the M337V variant (Arnold et al., 2013). A schematic diagram of the genome editing strategy used to introduce the Q331K point mutation into the *TARDBP* locus of human WTC11 iPSCs is provided in Figure 1A. Using this strategy, a cytosine base was replaced with an adenine, resulting in a precision point mutation of Glutamine to Lysine at position 331 (CAG- > AAG) in the translated amino acid sequence. This was confirmed by Sanger sequencing of the target site (Figure 1B). TDP-43^Q331K+/–^ heterozygous clones were selected for subsequent expansion to facilitate direct comparisons with the commercial lines and to increase the clinical relevance of any phenotypic observations as familial ALS patients typically harbor heterozygous disease-causing mutations in their genomes. All selected clones were karyotyped prior to further expansion and were found to bear normal XY karyotypes (Figure 1C).

Human iPSC cultures were differentiated into regionally unspecified neural progenitor cells using a monolayer differentiation method adapted from Shi et al. (2012). These cells were subsequently exposed to culture conditions promoting differentiation toward a ventral, spinal phenotype, essentially as described by Amoroso et al. (2013). This protocol led to the development of cells bearing a distinct neuronal morphology, characterized as multipolar cell bodies supporting extensive neuritic outgrowth (Figures 1D,E). These cells stained positive for the motor neuron-specific transcription factor Islet 1 (Figure 1F), indicating successful commitment to the motor neuron lineage. Immunocytochemical analysis of these cultures found that 63 ± 5.5% of the wild type population were positive for the pan neuronal marker microtubule associated protein 2 (MAP-2), whereas 32 ± 5.9% were positive for Islet-1. Similarly, populations derived from the TDP-43^Q331K+/–^ line were found to be 54 ± 3.0% positive for MAP-2 and 25 ± 5.4% positive for Islet-1. All Islet-1 positive cells examined also stained positive for MAP-2 in populations derived from both lines and observed differences in Islet-1 expression levels between mutant and control populations were not found to be statistically significant (Figures 1G,H).

**FIGURE 2:**
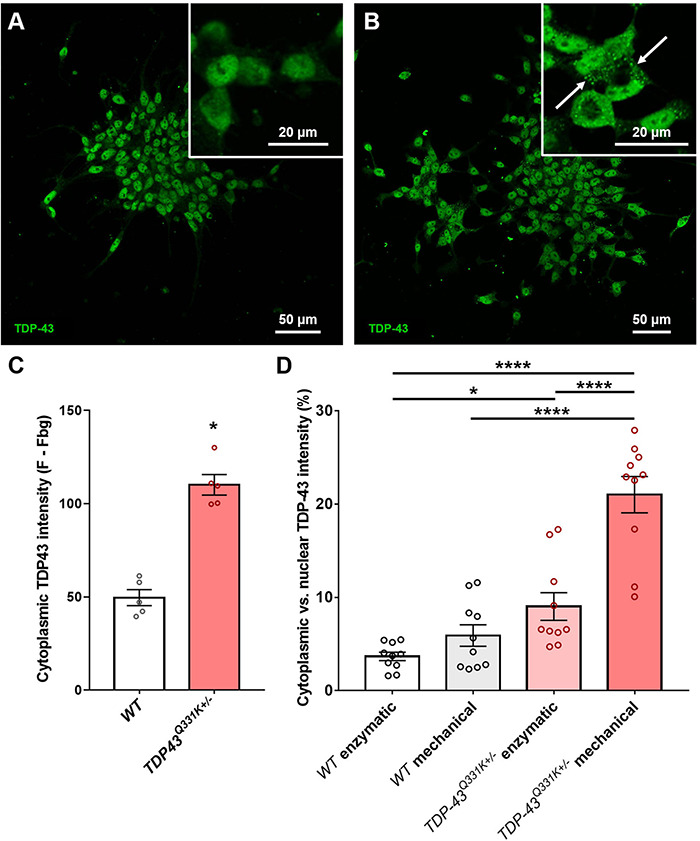
Quantification of TDP-43 cytosolic translocation in WTC11 wild type and *TDP-43*^Q331K+/–^ iPSC-derived motor neuron populations. **(A,B)** Immunocytochemical stains for TDP-43 in motor neuron populations differentiated in house from both wild type and *TDP-43*^Q331K+/–^ mutant WTC11 iPSCs. Mutant cells exhibited significant levels of cytoplasmic staining, indicating translocation of the protein from its normal nuclear location (white arrows). **(C)** Quantification of the comparative levels of cytoplasmic TDP-43 staining observed in mutant cells and wild type (*WT*) controls. **(D)** Levels of cytoplasmic TDP-43 (expressed as a percentage of signal intensity measured in nuclei) observed in wild type and mutant neurons exposed to either mechanical or enzymatic dissociation during differentiation. Analysis was performed at 25 days post-induction, with passaging performed at day 20. *p < 0.05, ****p < 0.0001.

Previous studies have shown that iPSC-derived motor neurons bearing ALS relevant *TARDBP* mutations exhibit significant levels of translocation of the TDP-43 protein from the normal nuclear location to the cytoplasm, associated with disruption of nuclear RNA splicing functions and with the formation of potentially toxic cytoplasmic aggregates (Winton et al., 2008; Zhang et al., 2009). This phenomenon resembles the TDP-43 proteinopathy reported in the vast majority of ALS patients and serves as an important indicator of correct disease recapitulation in cell-based models of the disease. In order to test whether the described CRISPR-edited WTC11 TDP-43^Q331K+/–^ line exhibited similar features, cells were stained and examined for TDP-43 subcellular localization. While all TDP-43 staining was restricted to the nucleus in wild type controls, mutant neurons displayed substantial levels of cytoplasmic staining and the emergence of distinct puncta indicative of protein aggregate formation (Figures 2A,B). Quantification of cytoplasmic intensity using ImageJ highlighted significant increases in TDP-43 positivity levels outside of the nucleus in TDP-43 mutant cells compared with wild type controls (Figure 2C).

Association of TDP-43 with cytoplasmic stress granules may contribute to seeding of cytoplasmic TDP-43 aggregation (Khalfallah et al., 2018) and oxidative stress likewise promotes cytoplasmic redistribution of TDP-43 (Zuo et al., 2021), suggesting that cellular stress may be an important determinant of TDP-43 redistribution. Our motor neuron differentiation protocol involves the replating of cells at multiple stages and relies on mechanical trituration to break up cell clusters during passage, leading us to ask whether the mechanical stress of our culture methods might influence cytoplasmic accumulation of TDP-43. To address this question, parallel differentiations were conducted using enzymatic dissociation with TrypLE instead of mechanical dissociation and the resulting levels of cytoplasmic TDP-43 were then quantified (Figure 2D). Results indicate that mechanical trituration during differentiation of iPSCs expressing mutant TDP-43 led to significant increases in TDP-43 aggregation. A small increase in cytoplasmic TDP-43 aggregation in mechanically triturated wild type controls was not found to bear statistical significance. Enzymatically dissociated mutant cells still exhibited a small but significant increase in cytoplasmic TDP-43 expression compared to wild type but this difference was substantially smaller than that observed in mechanically dissociated cells.

### Comparison of Splice Activity and Transcriptomes in Commercially Sourced and In-House Generated CRISPR-Edited Amyotrophic Lateral Sclerosis Lines

Bulk RNA-seq was performed on differentiated CDI and WTC11 populations. While presence of the Q331K mutation was confirmed for the WTC11 mutant line by Sanger Sequencing during its derivation, no similar confirmation had been performed for the commercial cells. We therefore began by confirming the presence of the M337V and Q331K mutations in the respective CDI lines prior to any downstream analysis using the raw RNA-seq reads (Supplementary Table 1).

Once presence of the mutations was confirmed in all lines, RNA-seq datasets were then used to compare and contrast the transcriptomes of TDP-43 wild type and mutant neurons derived from these sources (Supplementary Table 2). As expected, preliminary analysis of normalized expression values indicated that both CDI and WTC11 cultures exhibited strong expression of typical motor neuron markers such as Islet1 and choline acetyltransferase (ChAT). Conversely, expression of stem cell markers was notably absent, confirming the efficiency of the differentiation protocols employed for each cell type. Unsurprisingly, substantial expression of markers of V1, V2, and V3 ventral spinal cord interneurons were present in both CDI and WTC11 cultures, although the proportion of different interneuron classes differed. Both cultures were devoid of markers of astrocytes, but expressed substantial levels of an oligodendrocyte marker.

**FIGURE 3:**
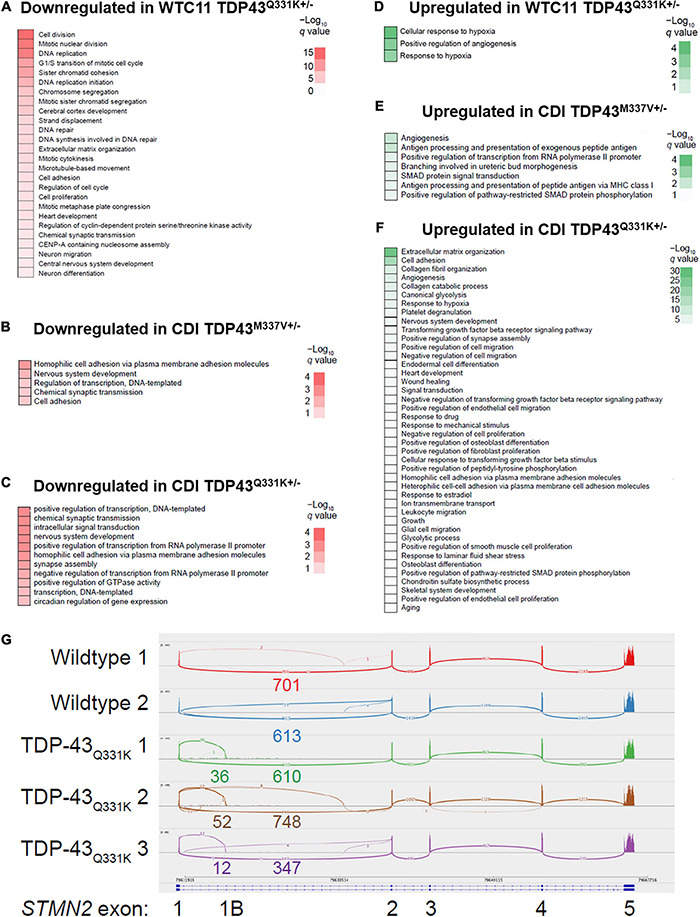
Effects of TDP-43 mutation on gene expression profiles and *STMN2* splicing behavior in CDI versus WTC11 motor neuron populations. **(A)** Gene ontology (GO) enrichment for genes downregulated in WTC11 TDP-43^Q331K+/–^ mutant samples compared with wild type controls. **(B)** GO enrichment for genes downregulated in CDI TDP-43^M337V+/–^ mutant samples compared with wild type controls. **(C)** GO enrichment for genes downregulated in CDI TDP-43^Q331K+/–^ mutant samples compared with wild type controls. **(D)** GO enrichment for genes upregulated in WTC11 TDP-43^Q331K+/–^ mutant samples compared with wild type controls. **(E)** GO enrichment for genes upregulated in CDI TDP-43^M337V+/–^ mutant samples compared with wild type controls. **(F)** GO enrichment for genes upregulated in CDI TDP-43^Q331K+/–^ mutant samples compared with wild type controls. **(G)** Integrated Genome Viewer Sashimi plot of splicing at STMN2. Three replicates of the TDP-43^Q331K+/–^ line demonstrate skipping to an intronic sequence.

Gene ontology (GO) biological process enrichment analysis was used to compare TDP-43 mutant cell lines for changes in cell signaling (relative to their control cell lines) (Figures 3A–F). WTC11 wild type and mutant lines exhibited significant differences in processes related to cell cycle, likely indicating differing levels of contaminating proliferating cells in the examined cultures. CDI cells showed no such contamination, thereby confirming the presence of a purer population. Despite this difference, consistent changes were observed, including down regulation of processes governing nervous system development in both CDI and WTC11 *TDP-43*^Q331K+/–^ mutants and upregulation of angiogenic and hypoxic processes across all mutant cell types examined. This consistency, despite differing levels of contaminating proliferating cells likely indicate these altered processes as significant in contributing to ALS cellular pathology in *TDP-43*^Q331K+/–^ mutant neurons. In the CDI lines, more biological processes were affected in the Q331K variant compared with M337V mutant cells; a result that likely reflects the stronger phenotype observed in Q331K mutant mice (Arnold et al., 2013).

Previous studies have indicated that ALS-associated TDP-43 mutations or TDP-43 proteinopathy associated with non-familial ALS cause mis-splicing of *STMN2* gene transcripts, splicing in a cryptic exon that results in greatly diminished steady-state levels of stathmin-2 mRNA and protein (Melamed et al., 2019). Consequently, we examined RNA-seq data for evidence of expression of the *STMN2* cryptic exon (Figure 3G). WTC11 *TDP-43*^Q331K+/–^ motor neurons expressed small amounts of the *STMN2* cryptic exon while wild type cells did not. Conversely, *STMN2* cryptic exon expression was absent in both *TDP-43^M337V+/–^* and *TDP-43*^Q331K+/–^ motor neurons from CDI (Supplementary Figure 2), highlighting a significant deviation in phenotype from both the WTC11 lines examined in this study and those discussed in previous work. These results were confirmed by RT-qPCR, which showed significant upregulation of transcripts bearing the cryptic exon in WTC11 mutant neurons compared with controls. Although these mutant neurons exhibited greater expression of transcripts bearing the cryptic exon, the proportion of such transcripts was not sufficient to diminish the total quantity of STMN2 mRNA expressed. As mis-splicing of the STMN2 cryptic exon is believed to result from redistribution of TDP-43 from the nucleus to cytoplasm (Prudencio et al., 2020), the lack of mis-splicing in the CDI neurons is consistent with lack of cytoplasmic TDP-43 observed in these cells.

### Comparison of Electrophysiological Phenotypes Between Commercially Sourced and In-House Generated CRISPR-Edited Amyotrophic Lateral Sclerosis Lines

Given the differences in transcriptome and *STMN2* splicing behavior between CDI and WTC11 motor neuron populations, electrophysiological characterization was sought as a means to compare functional development in these lines. Whole cell patch clamp was used to assess CDI cell action potential firing properties at 21 days post-thaw. This timepoint was selected as preliminary analysis indicated that a 3-week culture was necessary to promote the adoption of a mature electrophysiological phenotype in wild type controls (Supplementary Figure 3). In-house differentiated cells were examined at days 45–50 (and day 90) post-induction as initial studies indicated that length of culture was required for wild type controls to reach comparable functionality to that of their CDI counterparts.

**FIGURE 4:**
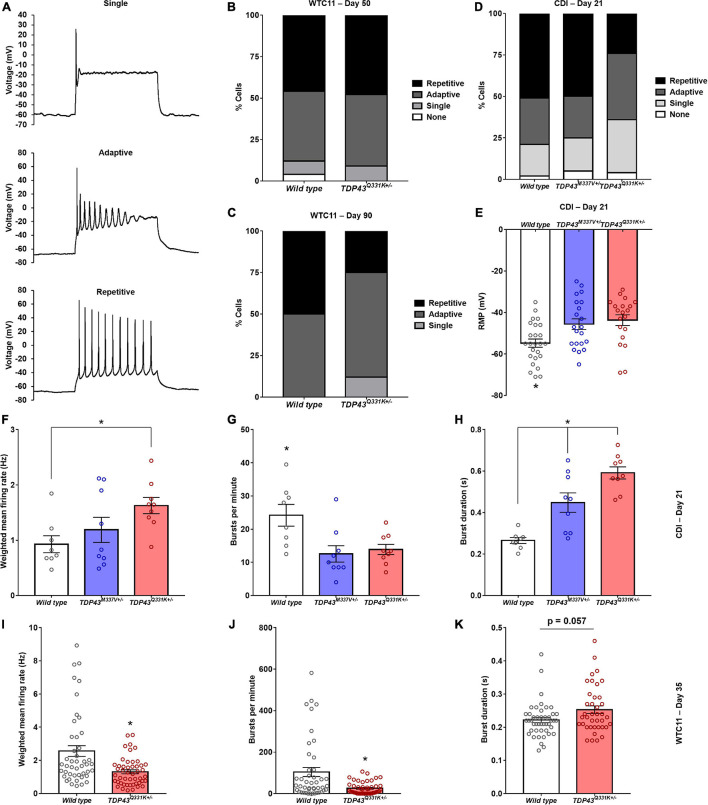
Electrophysiological assessment of WTC11 and CDI iPSC-derived motor neuron function. **(A)** Representative action potential firing properties recorded from CDI iPSC-derived motor neurons illustrating increasing functional maturation. **(B)** Percentage of WTC11 cells from wild type (*n* = 25) and TDP-43^Q331K+/–^ (*n* = 20) motor neuron populations exhibiting different action potential firing behaviors during whole cell patch clamp analysis at day 50 post-induction. A contingency table comparing cell type against firing behavior found that the two factors were not significantly related (*p* = 0.25), suggesting that frequency of firing type occurrence within each population is not dependent on the cell type examined at this timepoint. **(C)** Analysis of WTC11 motor neuron firing types at day 90 post-induction. At this time point, a contingency table comparing cell type against firing behavior found that the two factors were significantly related (*p* < 0.0001), suggesting that frequency of firing type occurrence within each population is dependent on the cell type examined. **(D)** Percentage of CDI cells from wild type (*n* = 43), TDP-43^M337V+/–^ (*n* = 20), and TDP-43^Q331K+/–^ (*n* = 25) motor neuron populations exhibiting different action potential firing behaviors during whole cell patch clamp analysis at 21 days post-thaw. A contingency table comparing cell type against firing behavior found that the two factors were significantly related (*p* = 0.0018), suggesting that frequency of firing type occurrence within each population is dependent on the cell type examined. **(E)** Resting membrane potentials measured from CDI wild type and mutant TDP-43 mutant motor neurons by whole cell patch clamp at 21 days post-thaw. *p ≤ 0.017. **(F)** Spontaneous rate of fire recorded from CDI motor neuron populations maintained on microelectrode arrays (MEAs). *p = 0.034. **(G)** Spontaneous burst fire behavior recorded from CDI motor neuron populations maintained on MEAs. *p ≤ 0.021. **(H)** Duration of spontaneous burst activity recorded from CDI motor neuron populations maintained on MEAs. *p ≤ 0.015. For panels **(F–H)**, the timepoint examined is 21 days post-thaw and *n* = 8 (wild type) or 9 (TDP-43^M337V+/–^ and TDP-43^Q331K+/–^). **(I)** Spontaneous rate of fire recorded from WTC11 motor neuron populations maintained on MEAs. *p = 0.0004. **(J)** Spontaneous burst fire behavior recorded from WTC11 motor neuron populations maintained on MEAs. *p = 0.0005. **(K)** Duration of spontaneous burst activity recorded from WTC11 motor neuron populations maintained on MEAs. *p = 0.057. For panels **(I–K)**, the timepoint examined is 35 days post-induction and *n* = 45 (wild type) or 48 (TDP-43^Q331K+/–^).

Cells were binned based on their capacity to elicit repetitive action potential trains in response to 500 ms depolarizing current injections as previously described (Devlin et al., 2015; Figure 4A). TDP-43^Q331K+/–^ WTC11 motor neurons showed comparable levels of function to wild type controls at day 50, with roughly 50% of patched cells exhibiting repetitive firing behavior (Figure 4B). Cells at day 90 showed a substantial decrease in repetitive activity in mutant cells compared with controls, indicating a progressive loss of function that mirrors previously reported results (Devlin et al., 2015; Figure 4C). In CDI cells, the TDP-43^M337V+/–^ line exhibited no significant change in firing patterns at day 21, whereas the TDP-43^*rm Q*331K+/–^ line displayed levels of function comparable to WTC11 mutant cells at day 90 (Figure 4D). In addition, both the M337V and the Q331K variants exhibited significantly more depolarized resting membrane potentials (RMPs) compared with wild type controls (Figure 4E); a hallmark indicator of poorer functional development and stability in cultured neurons. Specifically, average RMP in wild type cells was −54.8 ± 2.0 mV, compared with −45.6 ± 2.6 and −43.7 ± 2.6 mV in M337V and Q331K variants, respectively. No change in RMP was observed in WTC11 mutant cells at either day 50 or day 90 post-induction (Supplementary Figure 4).

Further characterization of TDP-43 mutant motor neuron electrophysiology was performed using multielectrode arrays (MEAs) in order to probe how altered action potential firing properties manifest at the population level. Following 21 days culture on MEAs (Supplementary Figure 5), CDI cells bearing M337V and Q331K mutations exhibited significant differences in population level function, with a notable decrease in burst fire behavior but an increase in burst duration and weighted mean firing rates (Figures 4F–H). Specifically, burst fire behavior was measured at 24.2 ± 3.3 bursts per minute (bpm) in wild type cells, 12.6 ± 2.5 bpm in M337V mutants, and 13.9 ± 1.5 bpm in Q331K populations. Burst durations were measured at 0.27 ± 0.01 s for wild type cells, 0.45 ± 0.05 s for M337V cells, and 0.59 ± 0.03 s for Q331K mutants. Overall weighted mean firing was recorded at 0.93 ± 0.15 Hz for wild type, 1.19 ± 0.23 Hz for M337V, and 1.63 ± 0.15 Hz for Q331K. A burst of neuronal activity is defined as a recording period where the time between detected depolarizing spikes is less than 100 ms for a minimum of 10 consecutive spikes (Chiappalone et al., 2005; Kapucu et al., 2012). Increased burst behavior is associated with maturing neuronal networks (Chiappalone et al., 2005) and a strong indicator of functional competency in cultured neurons. The observation that fewer bursts occurred in examined cultures, but that these events were longer once initiated, was noteworthy as it suggests an overall drop in spontaneous neuronal activation in mutant cells, but an increase in repetitive firing once activated, potentially mirroring hyperexcitability of ALS central and peripheral neurons in patients (Wainger et al., 2014; Noto et al., 2016; Do-Ha et al., 2018). WTC11-derived motor neuron populations exhibited a similar reduction in burst firing behavior in mutant cells (104.2 ± 21.9 bpm for wild type versus 25.5 ± 4.2 bpm for Q331K mutants) as well as a trend toward increased burst duration in the Q331K variants (0.22 s ± 0.008 for wild type and 0.25 ± 0.011 s for Q331K mutants; Figures 4I–K). Interestingly, weighted mean firing rate was reduced in WTC11 mutant cells but increased in CDI mutant lines compared with controls. This difference may reflect the significant difference in baseline firing rates between WTC11 and CDI control cells. Since the burst incidence rate was substantially higher in the WTC11 line, the significant reduction in burst activity observed in both mutant populations may have had a more drastic impact on the overall mean firing rate for the WTC11 cells than it did for the CDI neurons.

*In vivo*, spinal cord motor neurons synapse directly with skeletal muscle fibers and therefore should not form functional networks in culture. As such, the presented burst fire data likely indicate the presence of an interneuron population in the WTC11 cultures, which would facilitate network development and lead to the observed bursting behavior. Initial characterization of differentiated motor neurons revealed that the proportion of cells expressing the pan-neuronal marker MAP-2 was greater than the proportion of cells expressing the motor neuron marker, Islet-1, supporting this conclusion. Analysis of bulk RNA-seq data (discussed above) confirmed significant expression (relative to motor neuron-specific Islet1 expression) of V2a/V2b interneuron markers as well as lower expression of V3 markers in these cell populations.

### Analysis of Mitochondrial Structure and Metabolic Function in Commercially Sourced and In-House Generated CRISPR-Edited Amyotrophic Lateral Sclerosis Lines

To further compare and contrast the cellular phenotypes exhibited by CRISPR-edited TDP-43 mutant motor neurons from CDI and WTC11 sources, the structure and function of resident mitochondria were examined. Given the importance of metabolic dysfunction in contributing to ALS disease onset and progression (Tefera and Borges, 2017), the capacity for CRISPR-edited cell-based models of the disease to recapitulate this pathological feature is an important point to verify. Furthermore, GO biological process analysis of the collected RNA-seq data highlighted consistent alterations to pathways regulating hypoxia in both CDI and WTC11 neurons, underscoring the need to characterize mitochondrial phenotype in these cells.

**FIGURE 5:**
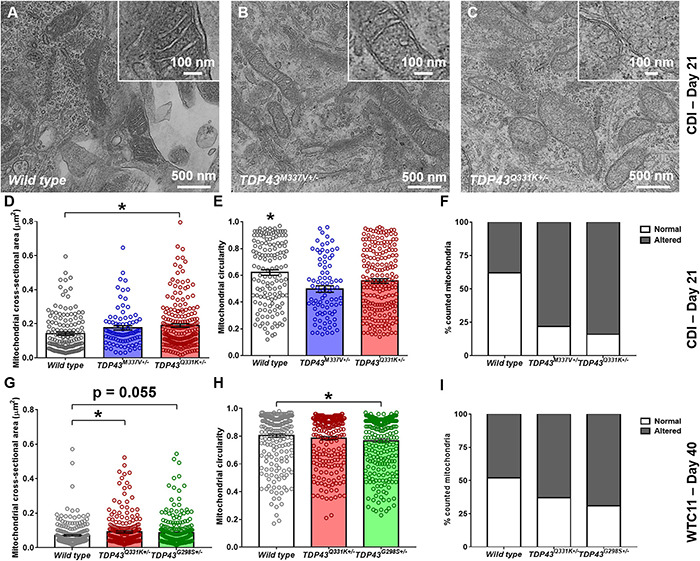
Analysis of mitochondrial structure in CDI and WTC11 wild type and TDP-43 mutant motor neurons. **(A–C)** Representative transmission electron micrographs (TEM) of mitochondria within CDI motor neurons. Insets illustrate details of cristae structure from mitochondria within each image. **(D)** Cross-sectional area measured from TEM images of mitochondria in CDI wild type (*n* = 144), TDP-43^M337V+/–^ (*n* = 85), and TDP-43^Q331K+/–^ (*n* = 202) motor neurons. Data from wild type and TDP-43^Q331K+/–^ samples were significantly different from each other (*p = 0.0016). **(E)** Circularity measured from TEM images of mitochondria in CDI wild type, TDP-43^M337V+/–^, and TDP-43^Q331K+/–^ motor neurons. Data from wild type neurons was significantly different from both TDP-43^M337V+/–^ and TDP-43^Q331K+/–^ samples (*p ≤ 0.037). **(F)** Percentage of mitochondria in CDI wild type (*n* = 43), TDP-43^M337V+/–^ (*n* = 20), and TDP-43^Q331K+/–^ motor neuron populations exhibiting normal and altered cristae structure, with normal being defined as regular, well defined cristae throughout the entire observable mitochondrial structure. A contingency table comparing cell type against cristae structure found that the two factors are significantly related (*p* = 0.0018), suggesting that frequency of normal cristae occurrence within each population is dependent on the cell type examined. **(G)** Cross-sectional area measured from TEM images of mitochondria in WTC11 wild type (*n* = 229) and TDP-43^Q331K+/–^ (*n* = 238) motor neurons and compared to results obtained from an ALS patient-derived iPSC line bearing a TDP-43^G298S+/–^ mutation (*n* = 361). Data from wild type and TDP-43^Q331K+/–^ samples were significantly different from each other (*p = 0.025), whereas data from wild type and TDP-43^G298S+/–^ patient samples were approaching significance (*p* = 0.055). **(H)** Circularity measured from TEM images of mitochondria in WTC11 wild type and TDP-43^Q331K+/–^ motor neurons and compared to results obtained from an ALS patient-derived iPSC line bearing a TDP-43^G298S+/–^ mutation. Data from wild type and TDP-43^Q331K+/–^ samples were not significantly different from each other (*p* = 0.0817), whereas data from wild type and TDP-43^G298S+/–^ mutant samples were (*p = 0.0035). **(I)** Percentage of mitochondria in WTC11 wild type and TDP-43^Q331K+/–^, as well as TDP-43^G298S+/–^ patient iPSC-derived, motor neuron populations exhibiting normal and altered cristae structure. A contingency table comparing cell type against cristae structure found that the two factors are significantly related (*p* = 0.0018), suggesting that frequency of normal cristae occurrence within each population is dependent on the cell type examined.

Transmission electron microscope (TEM) images were collected from wild type and TDP-43 mutant samples to characterize morphological differences in mitochondria resident within these cells (Figures 5A–C). In the CDI lines, a significant increase in cross-sectional area, along with a decrease in circularity, was observed in the mitochondria of mutant cells (Figures 5D,E). Specifically, average cross-sectional area in wild type CDI neurons was measured as 0.14 ± 0.009 versus 0.18 ± 0.012 and 0.19 ± 0.010 μm^2^ in M337V and Q331K variants, respectively. Circularity in wild type cells was measured as 0.62 ± 0.02 versus 0.50 ± 0.02 and 0.56 ± 0.02 in M337V and Q331K variants, respectively.

In addition to these readouts, mitochondria were binned as possessing “normal” or “altered” cristae structure based on the presence or absence of regularly spaced transverse protrusions throughout the entirety of the mitochondrial matrix (Figure 5F). Under these conditions, both M337V and Q331K variants exhibited a statistically significant increase in the number of observed “altered” cristae morphologies from examined cells. Together, these data suggest that TDP-43 mutations in iPSC-derived motor neurons lead to gross alterations in the size, elongation and cristae structure of resident mitochondria.

Similar results were observed in WTC11 motor neuron populations (Figures 5G–I), with wild type cells exhibiting an average cross-sectional area of 0.07 ± 0.004 μm^2^ compared with 0.09 ± 0.006 μm^2^ in the TDP-43^Q331K^ mutants. Circularity also decreased in WTC11 cell lines, going from 0.80 ± 0.01 μm^2^ in wild type cells to 0.78 ± 0.01 μm^2^ in the mutant line, although this difference was not statistically significant. These results highlight a consistency in mitochondrial phenotype between the two cell sources, albeit less pronounced in the in-house produced cells. Comparison of the WTC11 CRISPR-engineered neuron populations with cells derived from an ALS patient line bearing a *TDP-43^G298S+/–^* genotype further supported the notion that this alteration in mitochondrial structure was representative of TDP-43 mutant ALS. Specifically, the patient-derived cells showed a high degree of “altered” cristae morphologies, coupled with larger (cross-sectional area of 0.09 ± 0.005 μm^2^), less rounded mitochondria (circularity of 0.77 ± 0.01), largely recapitulating the phenotype observed in the CRISPR-edited WTC11 lines.

**FIGURE 6:**
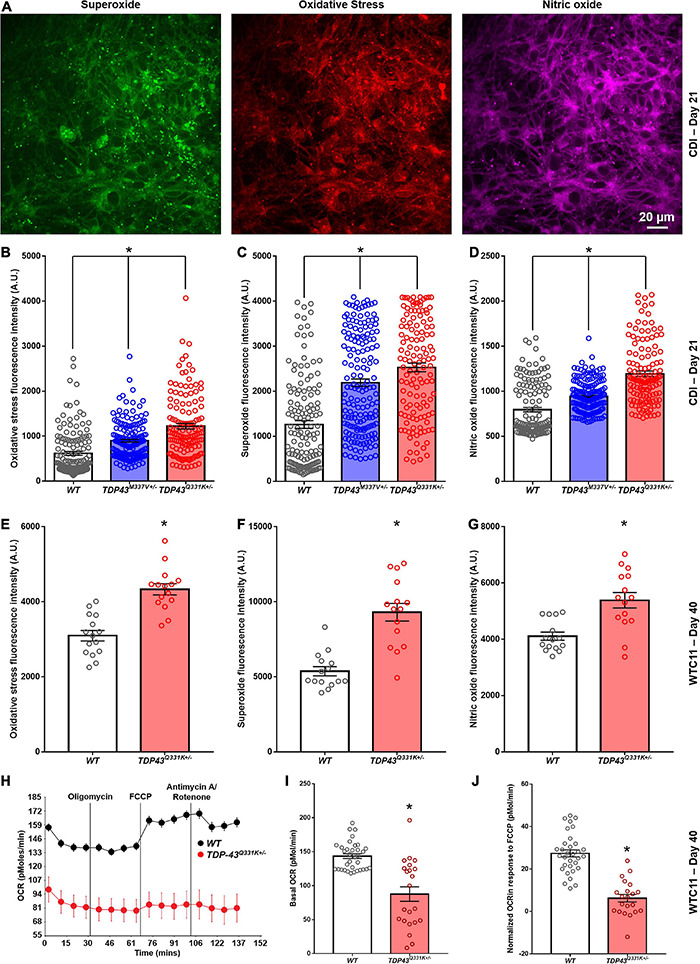
Analysis of reactive oxygen species production in CDI and WTC11 wild type and TDP-43 mutant motor neurons. **(A)** Representative staining from TDP-43^Q331K+/–^ mutant motor neurons illustrating expression of superoxide, nitric oxide, and markers of oxidative stress collected from CDI cells. **(B)** Levels of oxidative stress measured from CDI wild type (*n* = 276), TDP-43^M337V+/–^ (*n* = 304), and TDP-43^Q331K+/–^ (*n* = 262) motor neurons. All experimental groups were significantly different from each other (*p < 0.0001). **(C)** Levels of superoxide measured from CDI wild type, TDP-43^M337V+/–^, and TDP-43^Q331K+/–^ motor neurons. All experimental groups were significantly different from each other (*p ≤ 0.02). **(D)** Levels of nitric oxide measured from CDI wild type, TDP-43^M337V+/–^, and TDP-43^Q331K+/–^ motor neurons. All experimental groups were significantly different from each other (*p < 0.0001). **(E)** Levels of oxidative stress measured from WTC11 wild type (*n* = 15) and TDP-43^Q331K+/–^ (*n* = 15) motor neurons. *p < 0.0001. **(F)** Levels of superoxide measured from WTC11 wild type (*n* = 15) and TDP-43^Q331K+/–^ (*n* = 15) motor neurons. *p < 0.0001. **(G)** Levels of nitric oxide measured from WTC11 wild type (*n* = 15) and TDP-43^Q331K+/–^ (*n* = 15) motor neurons. *p = 0.0003. **(H)** Seahorse metabolic profiles measured from culture wells housing WTC11 wild type (*n* = 34) or TDP-43^Q331K+/–^ (*n* = 22) motor neurons. Measurements were taken at baseline and then following exposure to oligomycin, FCCP, and rotenone/antimycin A. **(I)** Basal oxygen consumption rate measured from WTC11 wild type (*n* = 34) or TDP-43^Q331K+/–^ (*n* = 22) motor neuron populations. *p < 0.0001. **(J)** Normalized oxygen consumption rate measured from WTC11 wild type or TDP-43^Q331K+/–^ motor neuron populations exposed to FCCP *p < 0.0001.

Mitochondrial dysfunction is often associated with increased production of reactive oxygen species (ROS). In view of the altered mitochondrial morphology associated with TDP-43 mutations, we performed analysis of ROS production in these lines using fluorescent dye probes. These results indicated that TDP-43 mutant cells exhibit higher relative expression levels of markers of oxidative stress, as well as superoxide and nitric oxide, than their wild type counterparts (Figures 6A–G). This increase was consistent between CDI and WTC11 cell sources and mirrors results reported previously for *C9orf72* mutant neuron lines (Lopez-Gonzalez et al., 2016). Based on the observed deficits in mitochondrial structure and function, Seahorse analysis was used to quantify oxygen consumption rates (OCRs) in these mutant motor neuron lines. Unfortunately, CDI cells were not able to survive the Seahorse assay protocol, possibly due to the purified nature of the cultures in question. WTC11 neuronal populations did survive and exhibited a significant drop in the basal OCR of *TDP-43*^Q331K+/–^ mutant cells, compared with wild type controls, as well as an additional reduction in OCR in response to FCCP exposure (Figures 6H–J). Specifically, measured basal OCR in wild type cells was 143.5 ± 3.5 and 87.6 ± 10.7 pMol/min in *TDP-43*^Q331K+/–^ mutant cells. OCR in response to FCCP exposure was 27.4 ± 1.7 pMol/min in wild type cells and 6.2 ± 1.8 pMol/min in *TDP-43*^Q331K+/–^ mutant neurons.

Taken together, these data highlight a significant reduction in oxidative metabolism in *TDP-43*^Q331K+/–^ mutant cells, coupled with an increase in ROS production, that correlates with altered cristae regularity and mitochondrial structure. Given the potential importance of these observations in adding to our understanding of cellular phenotypes in ALS, the RNA-seq data was interrogated for specific gene expression changes that may contribute to the highlighted mitochondrial phenotype. In data for the CDI motor neurons, the most heavily down-regulated gene for both M337V and Q331K variants was *CHCHD2*, a gene encoding the protein Coiled-Coil-Helix-Coiled-Coil-Helix Domain Containing 2. The downregulation of *CHCHD2* was confirmed in CDI cells *via* immunocytochemistry and RT-qPCR (Supplementary Figure 6). Remarkably, however, *CHCHD2* expression in the WTC11 motor neuron populations was found to be significantly upregulated in *TDP-43*^Q331K+/–^ mutant cells; the exact opposite result from that observed in CDI cells. A recent study found that a small chromosomal abnormality that frequently arises during expansion of human embryonic stem cells (20q11.21 amplification) causes loss of *CHCHD2* expression (Markouli et al., 2019). Such a culture artifact could potentially account for the diminished *CHCHD2* gene expression in the CDI TDP-43 mutant lines. This chromosomal abnormality is too small to be apparent in karyotypic analysis. However, 20q11.21 amplification is known to cause increased expression of *BCL2L1*, *ID1*, and *HM13*; three genes encoded at this locus. RNA-seq data revealed a modest increase in expression of *BCL2L1*, *ID1*, and *HM13* in the CDI TDP-43 mutant lines (data not shown) indicating that this 20q11.21 amplification may be present in these cells.

Loss of CHCHD2 activity has been reported to disrupt mitochondrial cristae structure (Zhou et al., 2018; Liu et al., 2020). However, as similarly abnormal mitochondrial structures were observed in two different *TDP-43*^Q331K+/–^ lines with oppositely altered levels of *CHCHD2* expression, the line-specific loss of *CHCHD2* expression does not appear to be responsible for the observed mitochondrial effects.

## Discussion

CRISPR-mediated gene editing, combined with cutting edge iPSC technology, holds significant potential for modeling human disease. Critical factors to consider when seeking to implement such methodologies for the study of disease etiology and progression is whether CRISPR-engineered phenotypes represent accurate depictions of the *in vivo* condition and whether these phenotypes are consistent regardless of the editing strategy employed. This study therefore sought to characterize the cellular phenotype of iPSC-derived motor neurons bearing CRISPR-engineered, ALS-relevant TDP-43 mutations. In addition, this work compared and contrasted the structural and functional differences that arose between TDP-43 mutant cells derived by different teams using distinct gene editing strategies. Critically, this comparison involved assessment of commercially sourced iPSC-derived neurons since such cells are typically used by drug companies for large-scale preclinical drug screens to reduce variability and enable normalization of results across sites (Scott et al., 2013).

Transcriptomic assessment of in-house derived and commercially sourced lines highlighted the relative purity and consistency of the commercial cells. While both CDI and WTC11 populations exhibited strong expression of motor neuron-specific transcripts, all these differentiated lines expressed significant levels of V1, V2 and V3 interneuron markers, and an oligodendrocyte marker, indicating the presence of a mixed population of ventral spinal neurons and supporting cells. Furthermore, although consistent levels of the motor neuron marker Islet1, substantial variance was observed in levels of ChAT expression, indicating a potential difference in the maturation state of motor neurons derived from these two sources. Variance in the number of contaminating proliferating cells between WTC11 wild type and *TDP-43*^Q331K+/–^ mutant populations also underscored the heterogeneity of the in-house differentiated lines. While methods for purifying iPSC-derived neurons from mixed differentiation runs have been described previously (Yuan et al., 2011), such techniques were not employed here as many of the assays employed in this study (Seahorse, MEA, etc.) required large numbers of cells and the attrition rates associated with antibody-based purification methods would have made such assays untenable. Despite differences in the base rate of fire and the associated rate of decline between CDI and WTC11 mutant populations, the consistent trends mirror results reported previously for patient-derived lines (Devlin et al., 2015), suggesting that the observed electrophysiological phenotype in these gene edited neuronal populations is representative of the human disease. Furthermore, the consistency of the observed electrophysiological dysfunction engenders confidence that this phenotypic aspect was not an artifact of the specific editing strategy used to create the mutant lines.

One of the hallmarks of ALS is mitochondrial dysfunction and some effort has been made to characterize the functional deficits of these organelles within iPSC-based models of *C9orf72*-mediated ALS (Lopez-Gonzalez et al., 2016). While mitochondrial defects in TDP-43 mutant neurons have been reported in rodent cells (Wang et al., 2013), to the best of our knowledge none have been reported previously in human neurons. RNA-seq results for both CDI and WTC11 mutant lines highlighted alterations in biological processes associated with regulating hypoxia, further underscoring the importance of characterizing metabolic behavior in these lines. TEM analysis revealed significant alterations in mitochondrial area and circularity, with mutant cells producing larger, more elongated organelles (suggesting increased fusion) with a significant reduction in the presence and regularity of cristae structures. This was coupled with increases in expression of oxidative stress markers and a substantial reduction in OCRs in TDP-43 mutant WTC11 neurons. Although similar increases in ROS production have been previously reported for *C9orf72* mutant cells (Lopez-Gonzalez et al., 2016), to the best of our knowledge, this study is the first to characterize the extent of mitochondrial dysfunction in human TDP-43 mutant neurons. Again, the consistency of phenotype observed between CDI and WTC11 populations, as well as the correlation of the collected data with results from patient-derived *TDP-43^G298S+/–^* cells, provides confidence in the relevance of the phenotype in terms of modeling ALS. However, results from the analysis of *CHCHD2* expression caution against over interpretation of the data. *CHCHD2* forms a heterodimer with closely related *CHCHD10*. This dimer is responsible for regulating cytochrome c oxidase activity within the inner mitochondrial membrane and regulating cristae structure. *CHCHD2* mutations are already associated with several neurodegenerative diseases, including frontotemporal dementia (Che et al., 2018) and Parkinson’s disease (Funayama et al., 2015; Meng et al., 2017), while *CHCHD10* mutations have also been found to cause ALS (Dols-Icardo et al., 2015; Zhang et al., 2015). As such, dysregulation of CHCHD2 in TDP-43 mutant cells could be a major contributor to ALS pathology in these patients. The CDI RNA-seq data seem to suggest that *CHCHD2* downregulation is one of the most profound impacts of TDP-43 mutation. However, previous work has indicated that *CHCHD2* is a gene that exhibits variable expression behavior across otherwise consistent iPSC lines (Zhu et al., 2016). Secondly, a number of studies based on iPSC models of neurodegenerative disease have reported *CHCHD2* mis-regulation and suggested this as a factor contributing to disease pathology (Shimojima et al., 2015; Xu et al., 2017; De Angelis et al., 2019). The emergence of altered *CHCHD2* expression in a variety of gene mutations responsible for dissimilar genetic diseases raises the red flag that such changes in gene expression may represent some stochastic and disease-unrelated epigenetic event associated with reprogramming and/or subcloning. Taking into account the available literature, it must be noted that the results reported here may likewise constitute an artifact of the subcloning associated with the generation of the gene edited CDI lines and not an actual effect of the mutation in the *TARDBP* gene. Such a hypothesis would seem to be supported by the WTC11 results, which conversely showed a significant upregulation of *CHCHD2* expression in the TDP-43 mutant line compared with isogenic controls.

The critical role of *CHCHD2* in regulating normal mitochondrial structure and function underscores the importance of comparing phenotypes across multiple lines when attempting to identify mechanisms contributing to ALS cellular characteristics. While the CDI results in isolation seem to highlight mis-regulation of *CHCHD2* as a potential contributor to cellular pathology in ALS, results from the WTC11 line indicate that such pathological features are present even without *CHCHD2* downregulation. As such, one can be relatively confident that the mitochondrial phenotype observed in this study represents a consistent feature of TDP-43 mutation in iPSC-derived neurons and may therefore be representative of ALS. This assumption is further supported by results from the patient-derived TDP-43^G298S+/–^ line, which confirm the presence of structural aberrations in the mitochondria of unedited mutant neurons. However, the role of *CHCHD2* mis-regulation in contributing to this defect requires further examination.

Extensive evidence points to expression of a cryptic exon in *STMN2* transcripts that may account for some features of the phenotype observed in cell-based models of ALS, such as diminished axonal regenerative capacity (Klim et al., 2019; Melamed et al., 2019). In the present study, alternative splicing of *STMN2* gene transcripts was observed in WTC11 *TDP-43*^Q331K+/–^ motor neurons but was notably absent in the comparable line from CDI. Furthermore, where present, the extent of STMN2 mis-splicing was not sufficient to reduce the overall level of STMN2 mRNA expression. Thus, stathmin-2 dysfunction is not responsible for the electrophysiological or mitochondrial abnormalities that were observed in both lines. All other aspects of phenotype characterized in this study showed consistency with in-house edited lines (that did show *STMN2* cryptic exon expression), indicating that at least some aspects of ALS cellular pathology can develop in cultured neurons in the absence of *STMN2* mis-splicing. These results serve to highlight the need for caution when interpreting the phenotypes of individual iPSC-lines as accurate models of disease and variations observed in *STMN2* splicing further underscore this point.

In this study, WTC11 mutant neurons exhibited significant TDP-43 translocation from the nucleus, in line with previous work (Winton et al., 2008; Zhang et al., 2009). Replacement of mechanical trituration with enzymatic dissociation led to significant reductions in the level of cytoplasmic TDP-43 expression observed. Since the level of cytoplasmic TDP-43 translocation would likely affect transcriptomic and splicing changes in the resulting cells, the provision of more gentle differentiation methods may reduce the phenotypic impact of the mutation by limiting stress granule formation. This observation raises the interesting possibility that aggregation of TDP-43 in ALS is stress dependent (Wolozin and Ivanov, 2019; Zuo et al., 2021), which might explain why ALS manifests as a late onset disease since it takes time for stresses to accumulate throughout life. This could also help explain why the clinical manifestations are so variable as they are dependent on the levels of environmental stress the patient is subjected to, which could vary significantly throughout an individual’s lifetime. Regardless, these data highlight that culture history can contribute to phenotype in iPSC-derived neuronal models of ALS, further underscoring the need to carefully dissect mutation-dependent effects from environmental cues when investigating disease mechanisms using such model systems.

In summary, commercial and in-house iPSC-derived motor neurons exhibited consistent electrophysiological phenotypes in TDP-43 mutant cultures. These results correlate with previously reported data from patient-derived cells, suggesting an accurate portrayal of functional decline in response to CRISPR-engineered mutations in *TARDBP*. Consistent dysregulation of mitochondrial structure and function was also observed across these mutant lines, indicating that degradation of cristae structure and increased ROS production occurs in response to TDP-43 mutation. While electrophysiological and mitochondrial abnormalities were consistently observed across all iPSC lines bearing *TARDBP* gene mutations, occurrence of *CHCHD2* mis-regulation and mis-splicing leading to *STMN2* cryptic exon expression showed line-specific variability, potentially due to differences in culture history and levels of mechanical stress during differentiation. These results indicate that TDP-43 mutant ALS possesses a complex cellular pathology that likely arises from multiple mechanisms, which reinforces the importance of confirming results in multiple lines to ensure accuracy when using CRISPR-based iPSC models to make predictions related to ALS disease mechanisms and/or drug responses.

## Materials and Methods

Unless otherwise stated, all incubation steps were carried out at 37°C/5% CO_2_.

### CRISPR-Mediated Introduction of *TARDBP* Mutation Into WTC11 Induced Pluripotent Stem Cells

Working with the University of Washington’s Institute for Stem Cell and Regenerative Medicine Ellison Stem Cell Core, heterozygous TDP-43^*Q*331*K*^ mutations were introduced into the WTC11 iPSC line, using methods described previously (Miyaoka et al., 2014). Cas9 guide RNAs (gRNA) targeting the *TARDBP* coding region were designed using a combination of web-based tools (CRISPoR and CRISPR-Scan websites). Particular attention was paid to the gRNA placement within the gene, off-target predictions, and SNP locations. To limit the exposure of the DNA to the genome editing enzyme and reduce off-target events, Cas9 protein and gRNA were introduced as ribonucleoprotein complexes. One million WTC-11 iPSC were electroporated with Cas9 (0.6 μM, Sigma) and gRNA (3 μM, Synthego) along with a ssDNA donor (2 μM, IDT) using Amaxa nucleofector (Human Stem Cell kit 2) in presence of ROCK inhibitor. Individual clones were handpicked and plated into 96 well plates. DNA was extracted using Quick Extract DNA extraction solution (Epicentre #QE09050) and nested PCR was performed using Forward (GCTTATTTTTCCTCTGGCTTTAGA) and Reverse (GATCCCCAACCAATTGCTGC) primers. The PCR product was purified using EXO-SAP enzyme (ThermoFisher) and sent for Sanger sequencing analysis (through Genewiz). Topo-cloning of PCR amplicons was performed for picked lines to validate their genotype. Clones harboring the Q331K mutation were amplified and sent for G-band karyotyping (outsourced to Diagnostic Cytogenetics Inc., Seattle, WA, United States).

gRNA sequence: GCAGCACTACAGAGCAGTTG

ssDNA sequence: GGTGGTGGGATGAACTTTGGTGCGTT CAGCATTAATCCAGCCATGATGGCTGCCGCCCAGGCAGC ACTAAAGAGCAGTTGGGGTATGATGGGCATGTTAGCCAG CCAGCAGAACCAGTCAGGCCCATCGGGTAATAACC.

### Cell Culture

Commercially sourced (iCell) human iPSC-derived motor neurons were purchased from CDI and were stored, thawed, and maintained according to the manufacturer’s protocol. Two different TDP-43 mutant lines were used in this study; M337V (CDI: CUS-MNC-1 × 01279.418) and Q331K (CDI: CUS-MNC-1 × 01279.435). In all experiments, the described mutant neurons were compared to an isogenic control iPSC-derived motor neuron line (CDI: R1049).

Culture surfaces were first treated with a 0.07% polyethylenimine solution (Sigma-Aldrich) and incubated overnight. Treated surfaces were then washed three times with sterile distilled water and allowed to dry for 1 h. Dried surfaces were treated with 5 μg/mL laminin (Sigma-Aldrich) and again incubated overnight. After incubation, the laminin solution was aspirated and cells plated immediately at 55,000 cells per cm^2^ using the medium provided by the manufacturer. Cultured motor neurons were fed every 2–3 days (50% medium replacement) and analyzed between days 20 and 22 of culture in all experiments described below.

WTC11 iPSCs (Kreitzer et al., 2013; Miyaoka et al., 2014) were passaged onto Matrigel-coated six-well plates and incubated at 37°C/5% CO_2_ in mTeSR until they reached ∼80% confluency. At this point, cultures were differentiated into regionally unspecified neural progenitor cells using a monolayer differentiation method adapted from Shi et al. (2012). These cells were then passaged onto 0.01% poly-L-ornithine (Sigma-Aldrich, St. Louis, MO, United States)/5 μg/mL laminin (Sigma-Aldrich)-coated surfaces and exposed to culture conditions promoting motor neuron differentiation, essentially as described by Amoroso et al. (2013). At days 20–25 post-induction, neurons were again passaged and replated onto final substrates (coated with poly-L-ornithine and laminin) for downstream experiments. In all experiments, cells were maintained in culture until they reached 40–50 days post-induction (or 90 days for certain patch clamp experiments) before being used in terminal analyses. All cells used in the described experiments were differentiated from WTC11 colonies between passage 45 and 55.

### RNA-Seq Analysis

Bulk RNA samples from WTC11 cells were isolated using the Trizol reagent and according to the manufacturers’ protocol. Quality of the isolated RNA was confirmed using TapeStation; only samples with an RIN value of 8.5 or greater were used for library preparation. Once RNA samples were isolated, library preparation was outsourced to the Fred Hutch Genomics Core (Seattle, WA, United States). Collection of raw RNA-seq data sets for the CDI lines was outsourced to Verge Genomics on behalf of the cell manufacturer and provided to the authors upon completion.

For both data sets, reads were aligned to the hg38 genome using TopHat (v.2.0.14) (Trapnell et al., 2010, 2012) to generate BAM files. Sequences were aligned to human mRNAs to generate FPKM values using Cufflinks v.2.2.1 (Trapnell et al., 2010, 2012) with the following parameters: “–library-norm-method quartile” and removing ribosomal, snoRNA and mitochondrial sequences. Differentially expressed transcripts (Jensen–Shannon divergence false discovery rate < 0.05) were obtained *via* Cuffdiff as part of the Cufflinks package. The overlap of differentially expressed genes between the two sets of samples was evaluated by the DAVID v6.7 GO program using a Benjamini *P*-value of <0.05 for significance (Huang Da et al., 2009a, b). WTC11 samples had between 120 and 130 million 50 bp paired-end reads. For TDP43 wild type versus Q331K, there were 753 downregulated and 782 significantly upregulated (*q* < 0.05 and log2fc > 1.5) genes, mapped to the hg38 refGene dataset. CDI samples had between 16.6 and 21.7 million 75 bp single-end reads. For CDI M337V versus control, 220 down-regulated and 254 upregulated genes were identified (*q* < 0.05 and log2fc > 0.5). For CDI Q331K versus control, 453 downregulated and 1062 upregulated genes were identified (*q* < 0.05 and log2fc > 0.5).

### Quantitative RT-PCR

RNA isolation from neuronal cultures was carried out using the Trizol reagent and according to the manufacturers’ protocol. cDNA synthesis was achieved using the iScript Reverse Transcription Supermix for RT-qPCR (Bio-Rad) and according to the manufacturer’s protocol. Quantitative RT-PCR (qRT-PCR) was carried out using the PrimePCR system provided by Bio-Rad. Ninety-six-well plates were ordered preloaded with primer pairs developed and validated by the manufacturer. The exception to this was the *STMN2* and truncated *STMN2* primers, which were derived from previously published work (Melamed et al., 2019). In this case, PCR products were run on a polyacrylamide gel, excised, and sent for sequencing to ensure the specificity of the primers used.

PCR reaction mixtures were prepared using the SsoAdvanced Universal SYBR Green Supermix with 500 ng of cDNA used per reaction. Plates were then run on a CFX96 real-time polymerase chain reaction (RT-PCR) detection system (Bio-Rad) and associated software using the thermal cycling protocol provided with the SsoAdvanced Universal SYBR Green Supermix kit. Detection thresholds for data analysis were set at the base of the linear phase and the resulting Ct values were analyzed by relative quantification, using the 2^∧−ΔΔCt^ method (Livak and Schmittgen, 2001). Gene expression in both M337V and Q331K mutant neurons were normalized to expression levels of glyceraldehyde 3-phosphate dehydrogenase (GAPDH) and expressed relative to levels recorded in normal isogenic control cells.

### Immunocytochemistry

Cells were fixed in 4% paraformaldehyde for 15 min and blocked with 5% goat serum in PBS for 1 h at room temperature. Cells were then incubated with primary antibodies diluted in 1% goat serum in PBS overnight at 4°C. The next day, cells were washed three times with PBS. They were then incubated in a secondary antibody solution containing secondary antibodies diluted in 1% goat serum in PBS. Counterstaining was performed with VECTASHIELD containing DAPI (Vector Labs). Images were taken at the Garvey Imaging Core at the University of Washington’s Institute for Stem Cell and Regenerative Medicine using a Nikon A1 Confocal System on a Ti-E inverted microscope platform. Twelve-bit 1024 × 1024 pixel images were acquired with Nikon NIS Elements 3.1 software. Antibodies used in this study were as follows: mouse anti-neurofilament (1 in 500, Millipore), mouse anti-microtubule associated protein-2 (1 in 1000, Millipore), mouse anti-Islet-1 (1 in 10, DSHB), rabbit anti-microtubule associated protein-2 (1 in 1000, Millipore), rabbit anti-CHCHD2 (1 in 300, Proteintech), Alexafluor-594 conjugated goat-anti-mouse secondary antibody (1:200, Invitrogen), and Alexafluor-488 conjugated goat-anti-rabbit secondary antibody (1:200, Invitrogen). In addition to the described methods above, the flurometric cellular ROS detection assay (Abcam) was carried out using the stains provided in the kit and according to the manufacturer’s protocol.

### Transmission Electron Microscopy

Cells were fixed in 4% Glutaraldehyde in a sodium cacodylate buffer and stored at 4°C overnight. Fixed cells were washed 5 × 5 min in 0.1 M cacodylate buffer, then post-fixed in osmium ferrocyanide for 1 h on ice. Cells were next washed 5 × 5 min in ddH_2_O and then incubated in a 1% thiocarbohydrazide solution for 20 min at room temperature. Cells were then washed again 5 × 5 min in ddH_2_O before being placed in 2% osmium tetroxide for 30 min at room temperature. Finally, cells were again washed 5 × 5 min in ddH_2_O before being en bloc stained in 1% uranyl acetate (aqueous) overnight at 4°C.

The next day, cells were washed 5 × 5 min in ddH_2_O, then en bloc stained in Walton’s lead aspartate for 30 min at 60°C. Following five more 5-min washes in ddH_2_O, cells were dehydrated in ice cold 30, 50, 70, and 95% EtOH, then allowed to come to room temperature. This was then followed by two changes of 100% EtOH and two changes of propylene oxide. Then, cells were infiltrated in a 1:1 mixture of propylene oxide: Durcupan resin for 2 h, followed by overnight infiltration in fresh Durcupan. The next day, cells were given a fresh change of Durcupan for 2 h and then placed in flat embedding molds and polymerized in a 60°C oven for 2 days. Eighty nanometers sections were then cut using a Leica EM UC7 ultra microtome and imaged on a JEOL 1230 TEM, at 80 kV.

### Image Analysis

Assessment of CHCHD2 and ROS staining intensity from confocal images of normal and TDP-43 mutant cells was performed using ImageJ. Similarly, measurement of mitochondrial size and circularity from TEM images was carried out in ImageJ. Assessment of gross mitochondrial structure was performed manually as previously described (Costa et al., 2018). Briefly, 30 micrographs were randomly taken for each cell type, and at least 100 mitochondria were counted and labeled either “normal” or “altered.” Normal mitochondria were defined as exhibiting organized and parallel cristae that ran perpendicular to each mitochondrion’s primary axis. Only cells observed with cristae spaced throughout the entire mitochondrial matrix were counted as normal to ensure consistency of analysis. Mitochondria that could not match these criteria exactly were placed in the “altered” group. All image analysis was blinded to remove bias.

### Electrophysiology

Whole-cell patch clamp recordings were performed on the 37°C heated stage of an inverted DIC microscope (Nikon) connected to an EPC10 patch clamp amplifier and computer running Patchmaster software (HEKA). Coverslips supporting cultured motor neurons were loaded onto the stage and bathed in a Tyrode’s solution containing 140 mM NaCl, 5.4 mM KCl, 1.8 mM CaCl_2_, 1 mM MgCl_2_, 10 mM glucose, and 10 mM HEPES. An intracellular recording solution containing 120 mM L-aspartic acid, 20 mM KCl, 5 mM NaCl, 1 mM MgCl_2_, 3 mM Mg^2+^-ATP, 5 mM EGTA, and 10 mM HEPES was employed and borosilicate glass patch pipettes (World Precision Instruments) with a resistance in the range of 3–6 MΩ were used for all recordings. Offset potentials were nulled before formation of a gigaΩ seal and fast and slow capacitance was compensated for in all recordings. Membrane potentials were corrected by subtraction of a 15 mV tip potential, calculated using the HEKA software. Cells that required more than 100 pA of current to achieve a −70 mV resting membrane potential were excluded as excessive application of current is indicative of poor patch quality and/or membrane integrity.

To generate a single action potential, a 5 ms depolarizing current pulse of sufficient intensity (1–2 nA) was applied. Depolarization-evoked repetitive firing was achieved *via* application of a series of 500 ms current injections starting at −30 pA and increasing in 10 pA increments. Both single action potentials and repetitive firing behavior were recorded in current-clamp mode. Inward and outward currents were evoked in voltage-clamp mode *via* a series of 500 ms depolarizing steps from –120 to +30 mV in 10 mV increments. Gap-free recordings of spontaneous activity in patched neurons were performed in current-clamp mode for 30 s with 0 pA current injection to provide a measure of the maximum diastolic potential held by the cell without current input. All recordings and analyses of action potential waveforms and currents were performed using the Patchmaster software suite.

Population level function in motor neuron cultures was assessed in 48-well MEA plates using the Maestro MEA system (Axion Biosystems). During data acquisition, standard recording settings for spontaneous neuronal spikes were used (Axis software, version 2.5), and cells were maintained at 37°C/5% CO_2_ throughout the 2-min recording period. The standard settings have 130× gain, and record from 1 to 25,000 Hz, with a low-pass digital filter of 2 kHz for noise reduction. In all experiments, spike detection was set at 5× the standard deviation of the noise and network burst detection was recorded if at least 25% of the electrodes in a given well showed synchronous activity. Reported results were calculated by averaging all of the electrodes in each well, then averaging data from duplicate wells.

### Oxygen Consumption Measurement Using the Seahorse Cellular Flux Assay

Seahorse assays were performed essentially as previously reported (Hussein et al., 2020). WTC11 neurons were seeded onto 96-well Seahorse plates at 100,000 cells/well at day 25 post-induction and maintained until day 45. Culture medium was then exchanged for base medium [unbuffered DMEM (Sigma D5030)] supplemented with sodium pyruvate (Gibco, 1 mM) and 25-mM glucose for 1 h prior to the assay. Substrates and selective inhibitors were injected during the measurements to achieve final concentrations of glucose (2.5 mM), 4-(trifluoromethoxy) phenylhydrazone (FCCP, 300–500 nM), oligomycin (2.5 mM), antimycin (2.5 mM), rotenone (2.5 mM), palmitate (50 mM in BSA), BSA, and ETO (50 mM). The OCR values were normalized to the number of cells present in each well, quantified by hoechst staining (HO33342; Sigma-Aldrich). Changes in OCR in response to substrates and inhibitors addition were defined as the maximal change after the chemical injection compared to the last OCR value recorded before the injection.

### Statistical Analysis

All experiments were performed at least in triplicate, and repeated using 2–3 independent vials of cells for CDI cultures or independent differentiation runs for WTC11 neurons. Significant differences between groups were evaluated using unpaired *t*-tests for two conditions, or one-way ANOVA, with *post hoc* tests for multiple comparisons, for experiments with three or more groups. Mann–Whitney *U* tests and ANOVA on ranks were used to analyze the statistical significance of differences arising between sets of non-normally distributed data. For repetitive firing analysis and normal versus altered mitochondrial structure comparisons, contingency tables were constructed and used to run Chi-squared tests to determine whether the distribution of data was dependent on cell type. In all experiments, a *p*-value of less than 0.05 was considered significant. All statistical tests were performed using the GraphPad Prism statistics software.

## Data Availability Statement

The datasets presented in this study can be found in online repositories. The names of the repository/repositories and accession number(s) can be found below: ArrayExpress, accession no. E-MTAB-10666.

## Author Contributions

AS conducted the majority of the experiments and wrote the manuscript. CC assisted with motor neuron differentiation and performed analysis of TDP-43 cytoplasmic inclusions. JH and JM were responsible for engineering the Q331K mutation into the *TARDBP* locus using CRISPR-Cas9 gene editing. PV conducted the RNA-seq analysis. DM, B-OC, D-HK, and MB oversaw the project, provided feedback on collected data, and provided edits to the manuscript. All authors contributed to the article and approved the submitted version.

## Conflict of Interest

The authors declare that the research was conducted in the absence of any commercial or financial relationships that could be construed as a potential conflict of interest.

## Publisher’s Note

All claims expressed in this article are solely those of the authors and do not necessarily represent those of their affiliated organizations, or those of the publisher, the editors and the reviewers. Any product that may be evaluated in this article, or claim that may be made by its manufacturer, is not guaranteed or endorsed by the publisher.
