# Supplementary material for: Human Induced Pluripotent Stem Cell-Derived TDP-43 Mutant Neurons Exhibit Consistent Functional Phenotypes Across Multiple Gene Edited Lines Despite Transcriptomic and Splicing Discrepancies
Source: Front Cell Dev Biol. 2021 Sep 29;9:728707. doi: 10.3389/fcell.2021.728707 (PMC8511491; doi:10.3389/fcell.2021.728707)
Supplement: Supplementary file 1 [file Data_Sheet_1.docx]

**Supplementary materials**

**Human iPSC-derived TDP-43 mutant neurons exhibit consistent functional phenotypes across multiple gene edited lines despite transcriptomic and splicing discrepancies**

Alec S.T. Smith^1,2^, Changho Chun^3^, Jennifer Hesson^2,4^, Julie Mathieu^2,4^, Paul N. Valdmanis^5^, David L. Mack^1,2,3,6^, Byung-Ok Choi^7,8,9^, Deok-Ho Kim^3,10,11,12^*, and Mark Bothwell^1,2^*.

1. *Department of Physiology and Biophysics, University of Washington, Seattle WA 98195, USA*
2. *Institute for Stem Cell and Regenerative Medicine, University of Washington, Seattle, WA 98109, USA*
3. *Department of Bioengineering, University of Washington, Seattle, WA 98195, USA*
4. *Department of Comparative Medicine, University of Washington, Seattle, WA 98195, USA*
5. *Division of Medical Genetics, University of Washington, Seattle WA 98195, USA*
6. *Department of Rehabilitation Medicine, University of Washington, Seattle WA 98195, USA*
7. *Department of Neurology, Samsung Medical Center, Sungkyunkwan University School of Medicine, Seoul, Republic of Korea.*
8. *Stem Cell & Regenerative Medicine Institute, Samsung Medical Center, Seoul 06351, Republic of Korea*
9. *Department of Health Sciences and Technology, SAIHST, Sungkyunkwan University, Seoul 06351, Republic of Korea*
10. *Department of Biomedical Engineering, Johns Hopkins University, Baltimore, MD 21205, USA*
11. *Department of Medicine, Johns Hopkins University School of Medicine, Baltimore, MD 21205, USA*
12. *Department of Neurology, Johns Hopkins University School of Medicine, Baltimore, MD 21205, USA*

* Authors share corresponding authorship

*Persons to whom correspondence should be addressed:

| Dr. Deok-Ho Kim  Department of Biomedical Engineering  The Johns Hopkins University  Ross Research Building, 724B  720 Rutland Avenue  Baltimore, MD 21205  E-mail: dhkim@jhu.edu | Dr. Mark Bothwell  Department of Physiology and Biophysics  University of Washington  850 Republican Street  South Building, Room S420  Seattle WA 98109  Email: mab@uw.edu |
| --- | --- |

Short title: ALS phenotyping in iPSC-derived neurons

| Table S1: Table of wild type and mutant reads for the CDI Q331K and M337V variants plus the control line across 3 independent samples (a, b, & c) | | | | | |
| --- | --- | --- | --- | --- | --- |
| Sample | Q331 | K331 | M337 | V337 | % mut. |
| M337V_a | 41 | 0 | 37 | 20 | 35.09 |
| M337V_b | 25 | 0 | 23 | 23 | 50.00 |
| M337V_c | 25 | 0 | 18 | 29 | 61.70 |
| Q331K_a | 40 | 25 | 56 | 0 | 38.46 |
| Q331K _b | 26 | 19 | 55 | 0 | 42.22 |
| Q331K _c | 36 | 23 | 51 | 0 | 38.98 |
| Control_a | 48 | 0 | 59 | 0 | 0.00 |
| Control_b | 63 | 0 | 78 | 0 | 0.00 |
| Control_c | 62 | 0 | 75 | 0 | 0.00 |

| **Table S2: Expression levels of cell type markers determined by RNAseq** | | | | |
| --- | --- | --- | --- | --- |
| **Gene** | **Specifies** | ***WTC11_TDP43 wild type_*** | ***WTC11_TDP43 Q331K +/-_*** | ***CDI*** |
| CALB | V1a Renshaw interneuron | 6.0 | 0.35 | 1.6 |
| LMO4 | V2a interneuron | 8.7 | 9.5 | 10.6 |
| VSX2/CHX10 | V2a/V2b interneuron | 2.4 | 2.1 | 1.8 |
| SIM1 | V3 interneuron | 0.5 | 0.9 | 0.2 |
| Islet1 | Motor neuron | 19.6 | 18.2 | 22.7 |
| CHAT | Motor neuron | 8.4 | 8.8 | 0.57 |
| RBFOX3 (NeuN) | Pan-neuronal | 0.14 | 0.45 | 0.98 |
| AQP4 | Astrocyte | < 0.02 | 0.11 | 0.03 |
| GFAP | Astrocyte | < 0.02 | 0.02 | 0.02 |
| CNP | Oligodendrocyte | 16.6 | 23.7 | 1.9 |
| POU5F1 (Oct3) | Embryonic Stem Cell | < 0.02 | < 0.02 | < 0.02 |
| Normalized relative expression levels calculated from RNAseq data for “motor neuron” cultures differentiated from WTC11 cells or obtained from CDI. The RNAseq data for WTC11 and CDI cultures were generated and analyzed in different laboratories so the results are not exactly comparable. Data represent the mean of two or three replicate cultures. Statistical parameters are not calculated because of the small value of N, but replicates varied by less than 10 percent of the mean in all cases. | | | | |

**
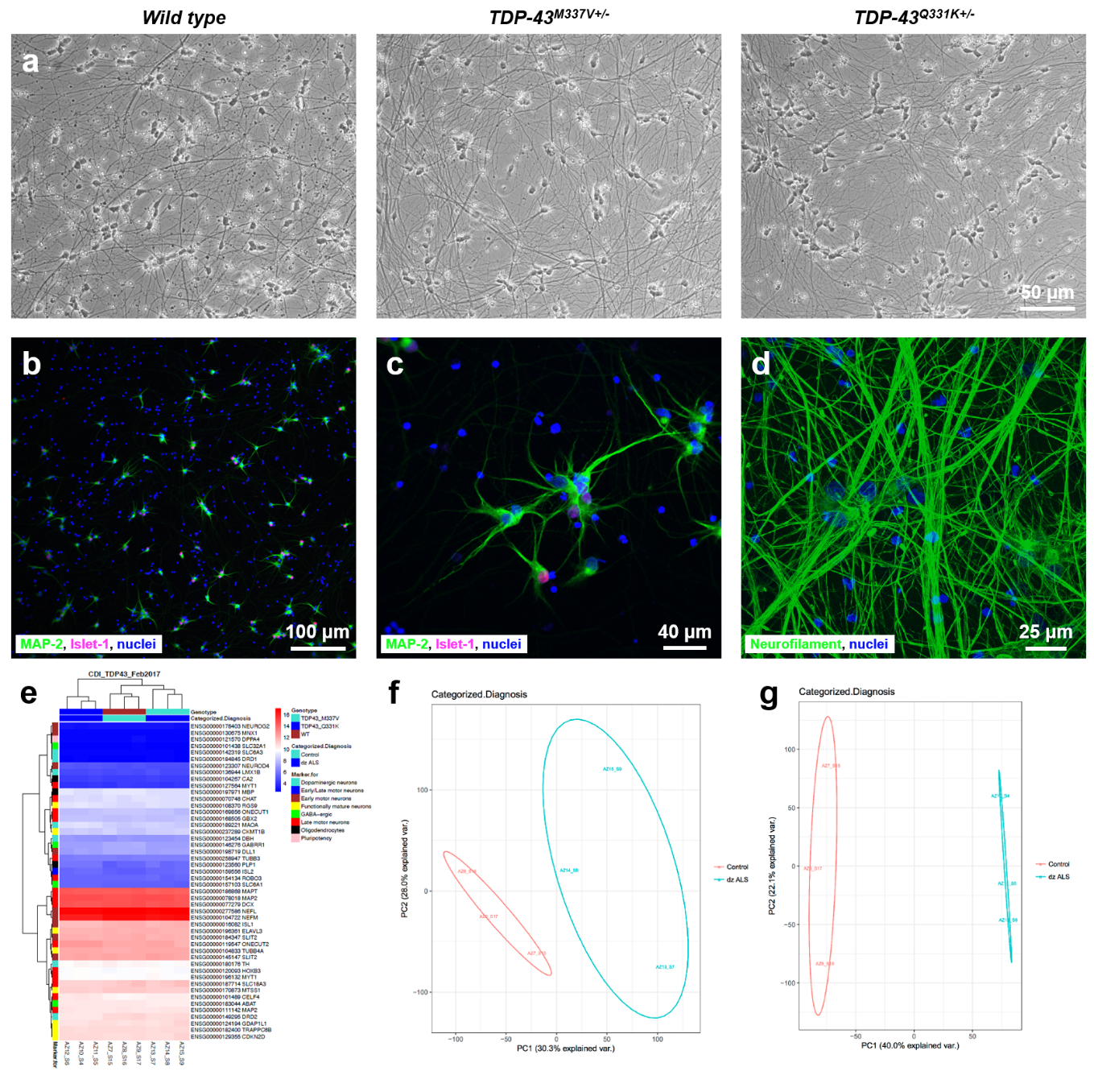
Figure S1. CDI motor neuron culture and phenotyping.** (a) Bright field images of wild type, TDP-43^M337V+/-^, and TDP-43^Q331K+/-^ mutant motor neurons derived from iPSCs by a commercial vendor (CDI). Images were collected at day 21 in culture. (b, c) Low (left) and high (right) magnification immunocytochemical images of commercially-sourced iPSC-derived motor neurons at day 21 stained for the pan neuronal marker MAP-2 and the motor neuron-specific transcription factor Islet-1. (d) High magnification immunocytochemical image of commercially-sourced iPSC-derived motor neurons at day 21 stained for the pan neuronal axon marker neurofilament. (e) Expression analysis of motor neuron markers from CDI wild type, TDP-43^M337V+/-^, and TDP-43^Q331K+/-^ motor neurons derived from RNAseq datasets. (f) PCA analysis of CDI wild type and TDP-43^M337V+/-^ motor neurons. (g) PCA analysis of CDI wild type and TDP-43^Q331K+/-^ motor neurons.

**
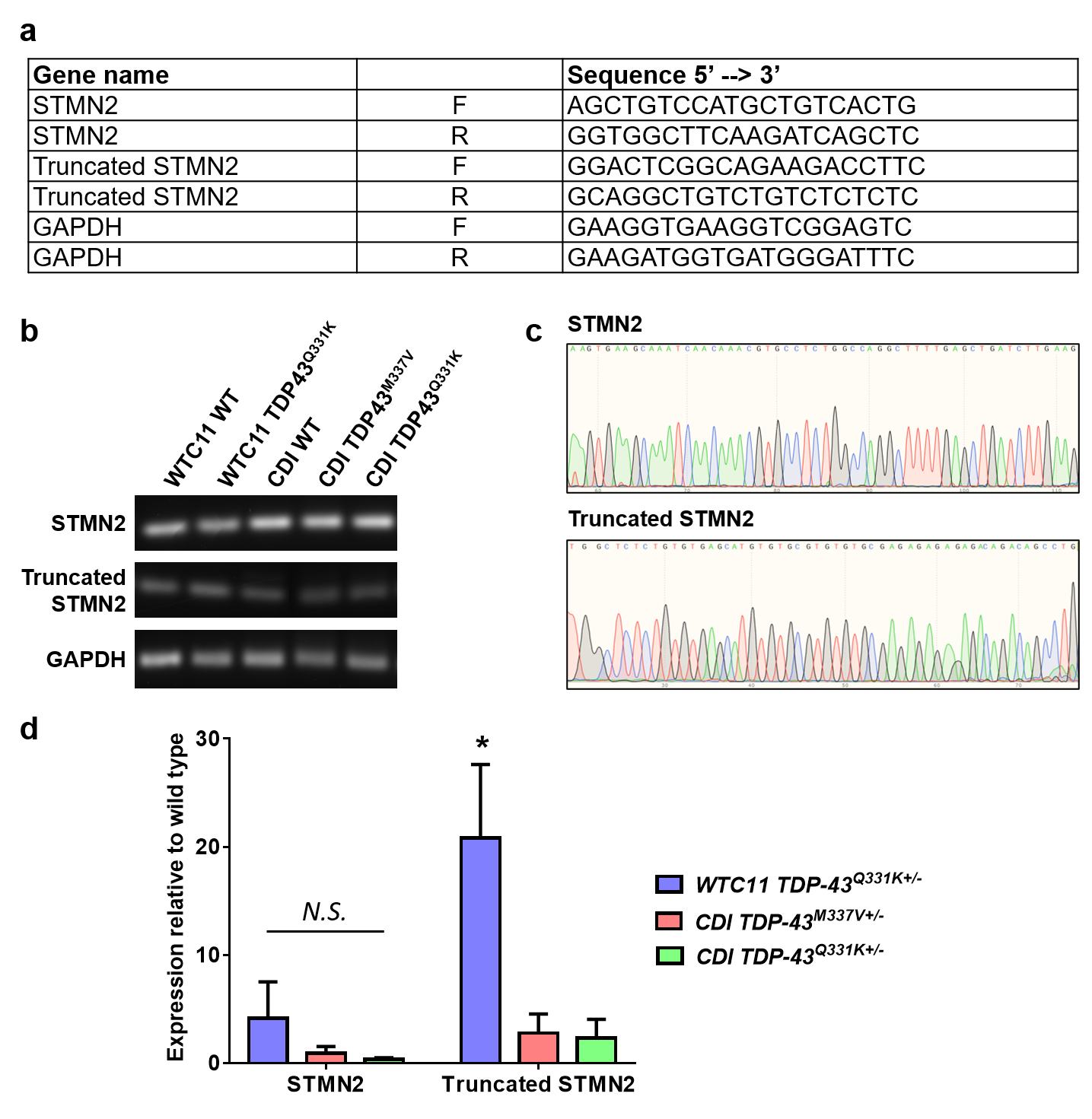
Figure S2. STMN2 splicing in WTC11 and CDI TDP-43 mutant motor neuron populations.** (a) Primers used to confirm STMN2 and truncated (cryptic exon) STMN2 expression in cultured neurons. Primers were drawn from previously published work (Melamed et al., 2019). (b) Polyacrylamide gel electrophoresis results showing production of a single band for each primer pair detailed in A. (c) Sequencing of PCR products illustrated in B. The sequence data was input into BLAST and confirmed to map to the STMN2 gene. (d) RT-qPCR results showing expression of full length and truncated STMN2 in TDP-43 mutant motor neuron populations. Presented data was normalized to GAPDH expression and expressed relative to wild type controls for each line examined. STMN2 expression levels were not significantly different (*N.S.*) between examined groups. Expression of the truncated *STMN2* transcripts were significantly higher (*p < 0.03) in WTC11 cells versus both CDI mutant variants.


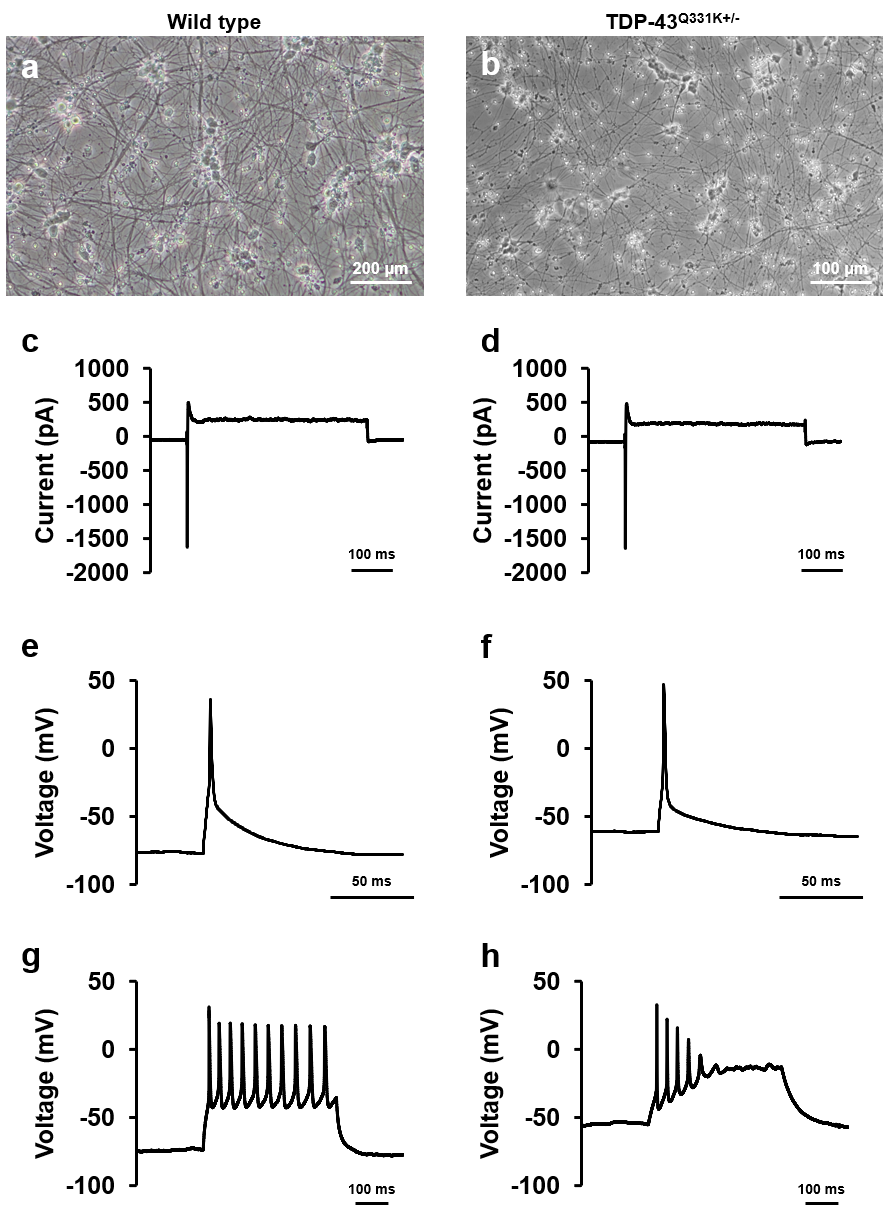


**Figure S3: Comparison of wild type and TDP-43 mutant CDI motor neuron electrophysiological profiles after 21 days in culture.** (a,b) Representative bright-field images of wild type and TDP-43^Q331K+/-^ CDI motor neurons at 21 days post-plating. (c,d) Representative voltage clamp recording from wild type and TDP-43^Q331K+/-^ CDI motor neurons at 21 days post-plating. (e,f) Representative single action potential recording in current clamp mode collected from wild type and TDP-43^Q331K+/-^ CDI motor neurons at 21 days post-plating. (g,h) Representative action potential firing behavior recorded in response to a 500 ms depolarizing current injection in current clamp mode from wild type and TDP-43^Q331K+/-^ CDI motor neurons at 21 days post-plating.


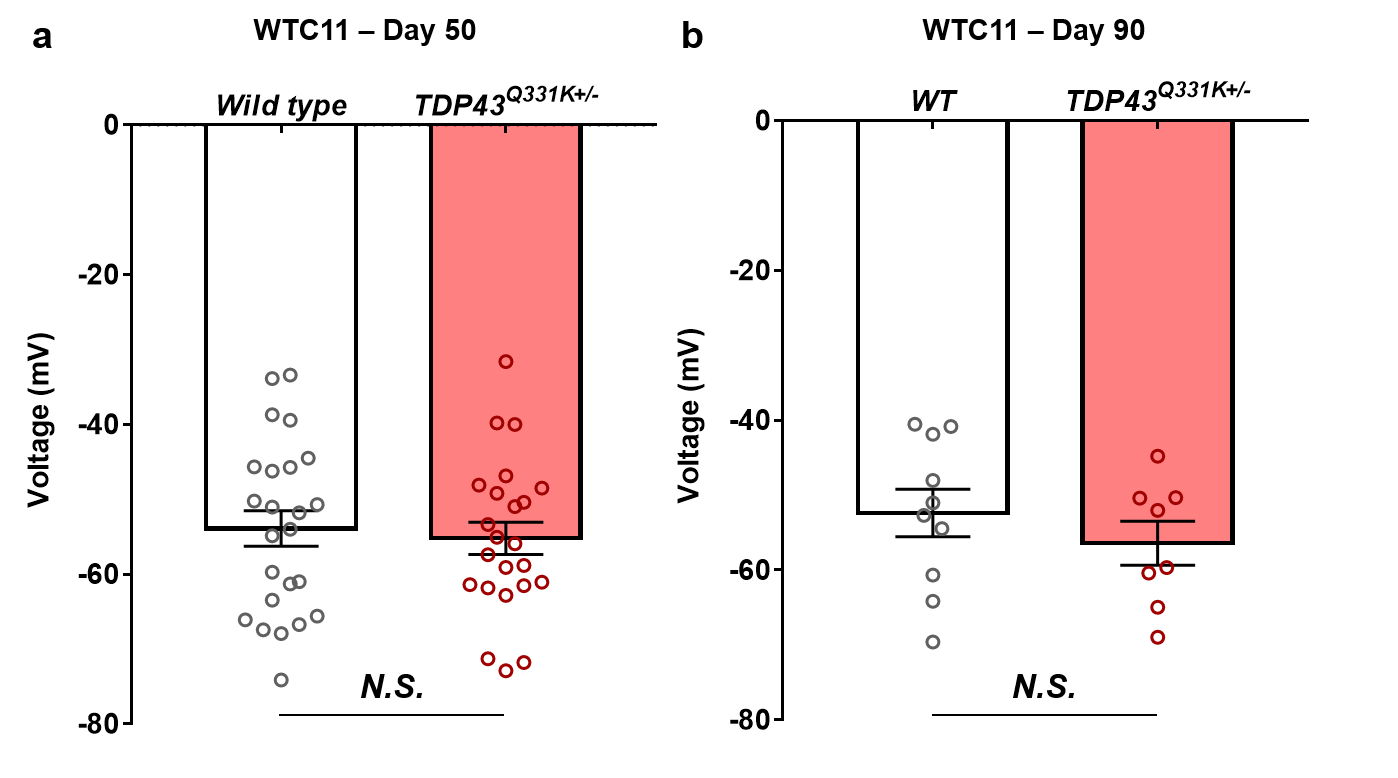


**Figure S4. Resting membrane potentials recorded from WTC11 motor neurons.** (a) RMP recordings from wild type (n = 24) and TDP-43^Q331K+/-^ mutant (n = 23) WTC11 motor neurons at day 50 post-induction (p = 0.78). (b) RMP recordings from wild type (n = 10) and TDP-43^Q331K+/-^ mutant (n = 8) WTC11 motor neurons at day 50 post-induction (p = 0.37).

**Figure S5: CDI motor neuron behavior on MEAs at different time points.** (a) Spontaneous rate of fire measured on MEAs and weighted for active electrodes. (b) Number of bursts recorded across a 2-minute period on MEAs. (c) Average burst duration measured on MEAs.


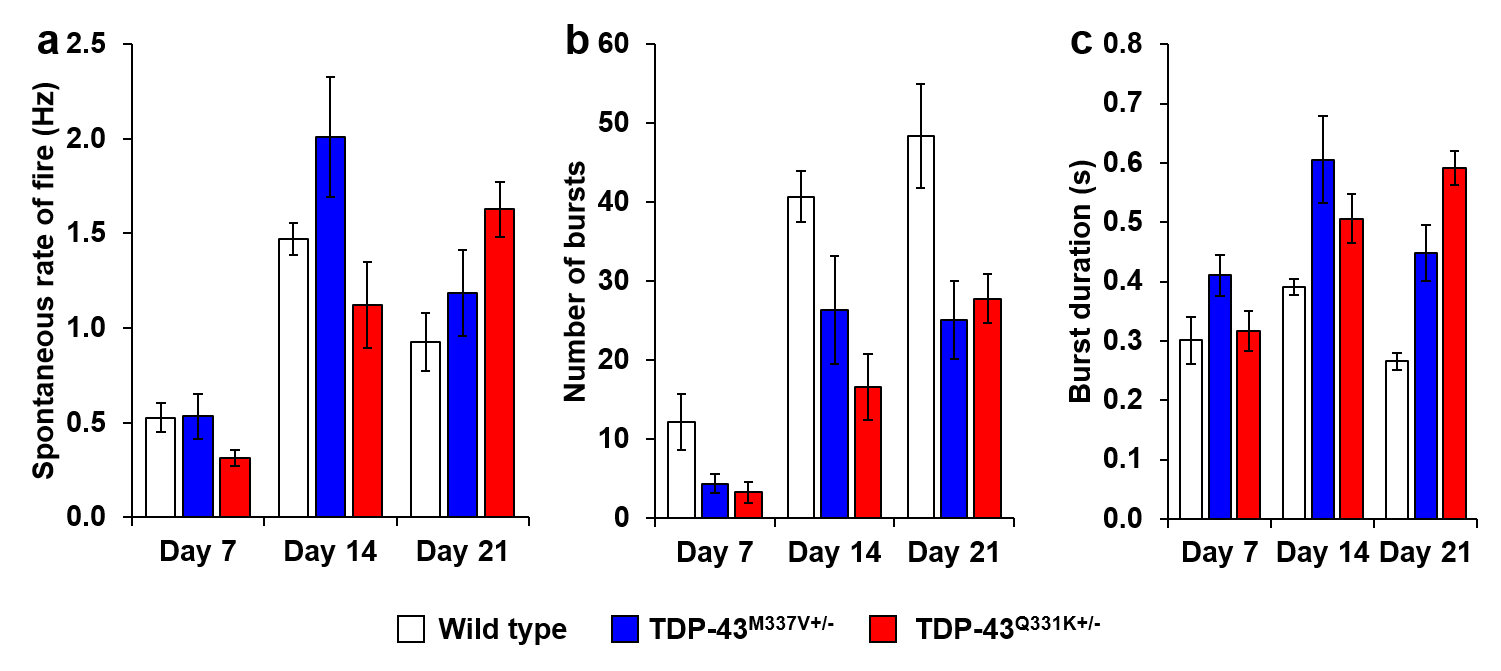


**
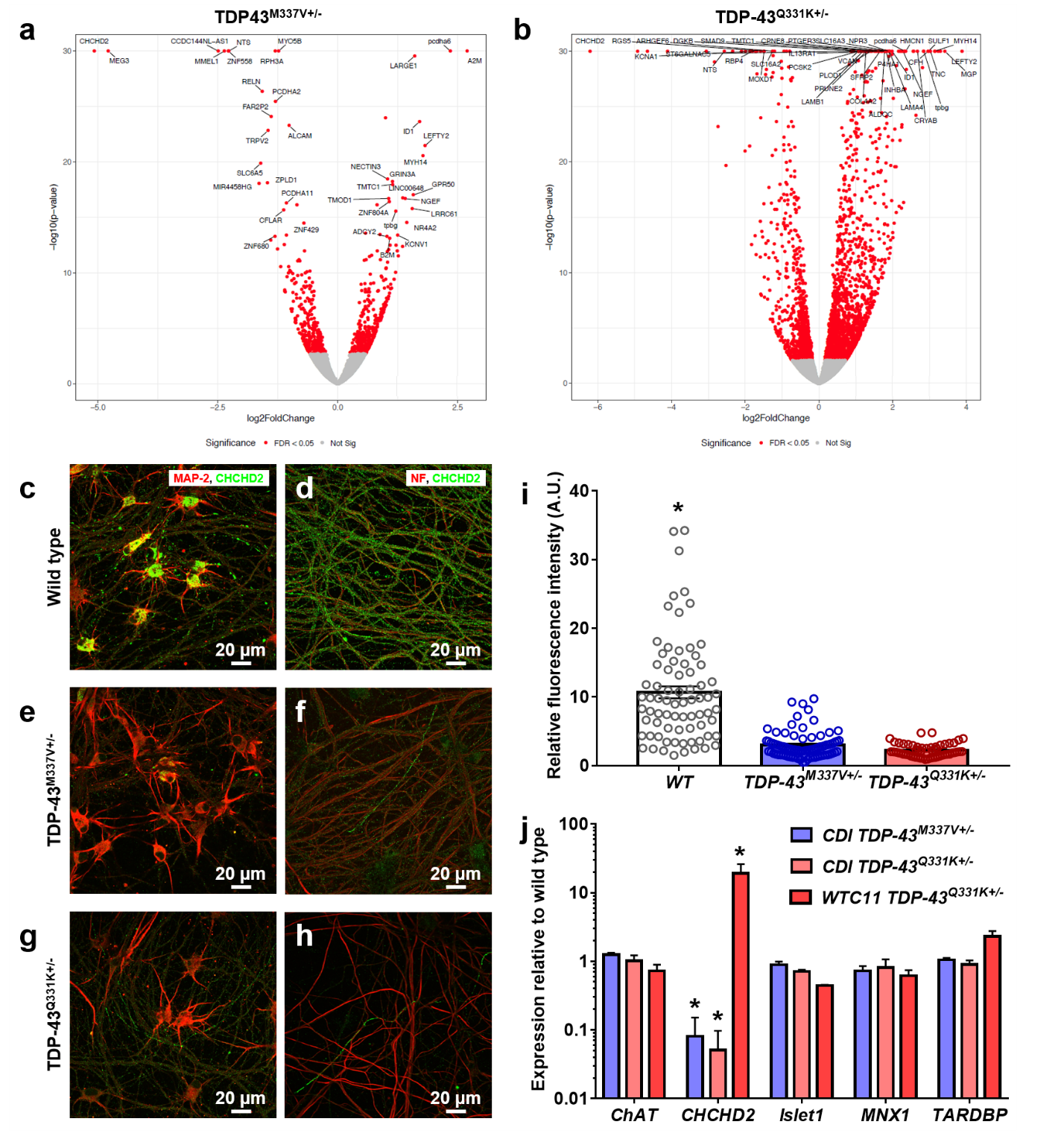
**

**Figure S6. CHCHD2 expression in CDI wild type and TDP-43 mutant motor neuron populations.** (a) Differential expression analysis in CDI TDP-43^M337V+/-^ mutant samples compared to wild type controls. (b) Differential expression analysis in CDI TDP-43^Q331K+/-^ mutant samples compared to wild type controls. (c,d) Representative immunocytochemical stains illustrating CHCHD2 expression in the cell bodies (c, co-stained with MAP-2) and neurites (d, co-stained with neurofilament) of CDI wild type motor neurons. (e,f) Representative immunocytochemical stains illustrating CHCHD2 expression in the cell bodies (e, co-stained with MAP-2) and neurites (f, co-stained with neurofilament) of CDI TDP-43^M337V+/-^ motor neurons. (g,h) Representative immunocytochemical stains illustrating CHCHD2 expression in the cell bodies (g, co-stained with MAP-2) and neurites (h, co-stained with neurofilament) of CDI TDP-43^Q331K+/-^ motor neurons. (i) Relative quantification of CHCHD2 expression calculated from fluorescence intensity measurements of MAP-2 positive cell bodies in CDI wild type (n = 74), TDP-43^M337V+/-^ (n = 81), and TDP-43^Q331K+/-^ (n = 55). Data collected from wild type cells was significantly different from both mutant lines examined (*p < 0.001). (h) RT-qPCR analysis of CDI and WTC11 TDP-43 mutant lines. Threshold fluorescence detection was normalized to an internal housekeeping gene (GAPDH) and expressed relative to levels detected in wild type control populations for each iPSC line examined. Expression of CHCHD2 was significantly different from the control value (1) across all mutant lines examined (*p < 0.001).
